# Discovery of NV-5138, the first selective Brain mTORC1 activator

**DOI:** 10.1038/s41598-019-40693-5

**Published:** 2019-03-11

**Authors:** Shomit Sengupta, Emilie Giaime, Sridhar Narayan, Seung Hahm, Jessica Howell, David O’Neill, George P. Vlasuk, Eddine Saiah

**Affiliations:** Navitor Pharmaceuticals, Inc., 1030 Massachusetts Ave. #410, Cambridge, MA 02138 USA

## Abstract

The mechanistic target of rapamycin complex 1 (mTORC1) has been linked to several important chronic medical conditions many of which are associated with advancing age. A variety of inputs including the amino acid leucine are required for full mTORC1 activation. The cytoplasmic proteins Sestrin1 and Sestrin2 specifically bind to the multiprotein complex GATOR2 and communicate leucine sufficiency to the mTORC1 pathway activation complex. Herein, we report **NV-5138**, a novel orally bioavailable compound that binds to Sestrin2 and activates mTORC1 both *in vitro* and *in vivo*. NV-5138 like leucine transiently activates mTORC1 in several peripheral tissues, but in contrast to leucine uniquely activates this complex in the brain due lack of metabolism and utilization in protein synthesis. As such, **NV-5138** will permit the exploration in areas of unmet medical need including neuropsychiatric conditions and cognition which have been linked to the activation status of mTORC1.

## Introduction

Sestrins are a small family of stress-inducible proteins that impact multiple biological processes including oxidative stress, the DNA damage response, metabolic homeostasis and mTORC1 signaling (reviewed in^1^). Overexpression of Sestrins in model organisms such as *Drosophila melanogaster* and *Caenorhabditis elegans* and in mammalian cells induce phenotypes similar to genetic or pharmacological inhibition of mTORC1 such as extending lifespan and maintaining metabolic homeostasis^2–5^. Invertebrates express a single Sestrin isoform while in mammals there are three Sestrin genes (*Sesn1/2/3*) that all negatively regulate mTORC1 signaling^6^. Initial reports that Sestrins inhibit mTORC1 via 5′ adenosine monophosphate-activated protein kinase (AMPK) were not consistent with the overexpression of Sestrin2 in mouse embryonic fibroblasts null for AMPK suggesting additional/alternative mechanisms for mTORC1 inhibition^7^. More recent studies have demonstrated that Sestrin2 directly modulates mTORC1 activation via a specific interaction with the multimeric complex GATOR2- part of the pathway that mediates the regulation of mTORC1 activation in response to the availability of amino acids^8–10^. Amino acid sufficiency results in recruitment of mTORC1 to the lysosomes via the heterodimeric RagA/B and RagC/D GTPases. The active state of RagA/B is modulated by the GAP activity of the protein complex GATOR1, which in turn is negatively regulated in an unknown manner by the large protein complex GATOR2 (reviewed in^11^). Sestrins are thought to directly bind and negatively inhibit GATOR2 thus, releasing its inhibition of the GAP-activity of GATOR1 resulting in inhibition of mTORC1. The inhibition of mTORC1 by Sestrin1 and Sestrin2 can be rapidly reversed by the influx of sufficient levels of amino acids, in particular, the branched-chain amino acid leucine whereas Sestrin3 is apparently not regulated by amino acids^10^. The presence of leucine results in the rapid disassociation of Sestrins 1 and 2 from GATOR2 restoring inhibition of GATOR1 and resulting in activation of mTORC1 signaling. Biophysical evidence indicates that leucine binds directly to a recombinant form of Sestrin2 and that leucine binding to Sestrin1 and 2 is required for disassociation from GATOR2^12^. Sequence analysis indicates high levels of conservation between the leucine binding site for Sestrin1 and Sestrin2 however, in contrast to Sestrin2, detailed biophysical studies using Sestrin1 have not been technically feasible due to the difficulty in obtaining a functional form of the recombinant protein. Thus, the detailed biochemical and biophysical work presented previously and herein focuses on Sestrin2 and assumes that the role of Sestrin1 is analogous. The discovery of leucine binding by Sestrin2 reveals two distinct mechanisms that modulate Sestrin2 binding to GATOR2. In the lack of cellular stress and under normal growth conditions, an equilibrium exists between Sestrin2 bound to leucine and Sestrin2 bound to GATOR2 allowing for mTORC1 modulation by fluctuations in leucine levels. However, in response to multiple stress-response pathways, protein levels of Sestrin2 increase via transcriptional regulation relative to GATOR2 driving the equilibrium towards more Sestrin2 bound to GATOR2 leading to inhibition of mTORC1^13^.

The pharmacological modulation of the mTOR pathway holds promise in a wide range of therapeutic indications and has almost exclusively centered on inhibitors with the identification of rapamycin and related derivatives (rapalogs)^14^ followed by the discovery of active site mTOR kinase inhibitors^15^. However, the discovery and utility of pharmacological activators of mTORC1 pathway signaling has remained unexplored. Suppressed mTORC1 signaling has been mechanistically linked to diseases such as major depressive disorder (MDD) and retinitis pigmentosa^16,17^. Multiple literature reports have demonstrated that the therapeutic efficacy of NMDA receptor modulators such as ketamine and rapastinel in animal models of depressive behavior is dependent upon mTORC1 activation in the areas of the brain responsible for mood such as the medial pre-frontal cortex^16^. Although ectopic activation of mTORC1 in peripheral tissues can be accomplished by administering a large dose of exogenous leucine, mTORC1 signaling in the CNS remains refractory likely due to the rapid turnover and buffering of leucine in the brain^18,19^. The discovery of Sestrin1 and 2 as a specific leucine sensor upstream of mTORC1 provided a unique opportunity to develop drug-like small molecule compounds that selectively activate mTORC1 pathway signaling via the binding to this leucine sensor. Herein, we describe the novel brain-penetrant Sestrin2-binding ligand **NV-5138** which is capable of mediating the activation of mTORC1 pathway signaling *in vitro* and *in vivo*. Oral administration of **NV-5138** transiently activates mTORC1 pathway signaling in the brain of *ad-libitum* fed rats in addition to several peripheral tissues. The activation of mTORC1 pathway signaling in the brain following oral administration differentiates **NV-5138** from leucine and correlates with high exposure of **NV-5138** in the brain and lack of metabolism and proteinogenic capacity. The specificity, drug-like properties and high CNS penetrance of **NV-5138** make it an ideal compound to evaluate in CNS diseases linked to reduced mTORC1 pathway activation including depression, and conditions linked to cognition, learning, and memory.

## Results

### Detection of Sestrin1 and Sestrin2 mRNA in neurons

Prior to initiating our efforts to develop CNS-active mTORC1 activators via Sestrin1/2 binding, we first wished to confirm mRNA expression of both sensors in the neurons in the brain. While previous publications have detailed a role for Sestrin2 in neuronal function^20–22^, Sestrin1 expression in neurons has not been firmly established. Sestrin1 is predicted to have two isoforms while Sestrin2 is predicted to have only one isoform^10^. Using RNA probes that recognize either both isoforms of Sestrin1 or Sestrin2 (red) in combination with a RNA probe recognizing the neuronal marker NeuN (turquoise), we performed RNA *in situ* hybridization on coronal brain slices from *ad libitum* fed male Sprague Dawley rats. The results clearly indicate both Sestrin1 and Sestrin2 are expressed in neurons throughout the brain including in the medial prefrontal cortex (Supplementary Fig. 1a). Sestrin1 expression was higher than Sestrin2 most likely due to detection of both isoforms (Supplementary Fig. 1a). Specifically, expression of Sestrin1 and Sestrin2 was found in neurons of the medial prefrontal cortex, hippocampus, striatum, and cerebellum among areas surveyed (Supplementary Fig. 1a). This data confirms previously published findings detailing neuronal localization of Sestrin2 and uncovers robust expression of Sestrin1 as well; thus, supporting the goal of developing CNS-active mTORC1 activators via targeting the Sestrin1/2 pathway.

### Design of NV-5138

**Figure 1:**
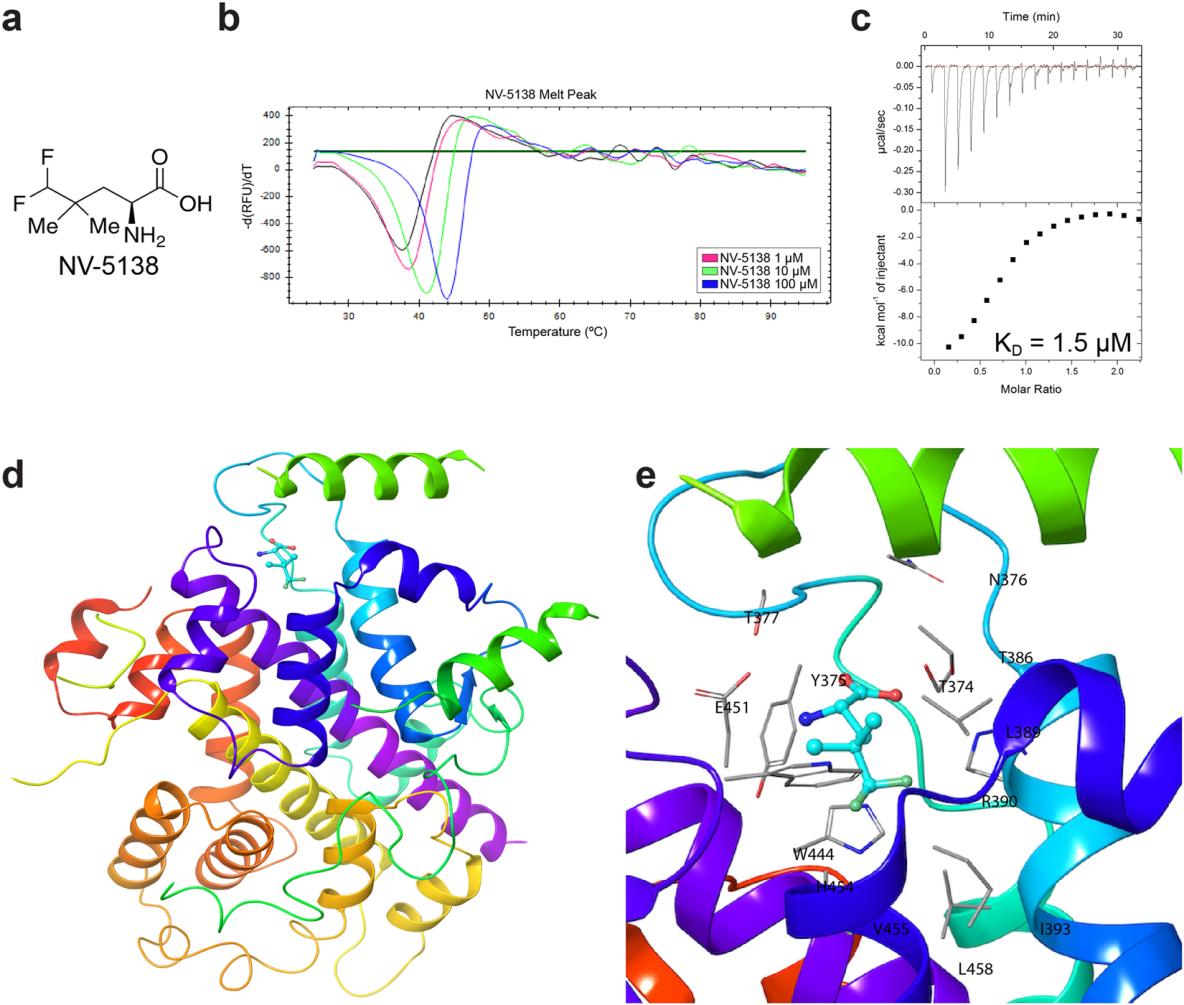
NV-5138 is a novel leucine analog that binds the leucine-binding pocket of Sestrin2. (**a)** Chemical structure of **NV-5138**. (**b**) Melt curve of Sestrin2 in the absence and presence of increasing amounts of **NV-5138**; pink = 1 µM, green = 10 µM, blue = 100 µM. (**c**) Measurement of the binding affinity of **NV-5138** for Sestrin2 by isothermal calorimetry (ITC) predicts a binding K_d_ of 1.5 µM with a molar stoichiometry of 1. (**d**) X-ray crystal structure of **NV-5138** bound to sestrin 2 at 3.3 Å resolution. (**e**) Interactions made by **NV-5138** in the leucine-binding pocket of sestrin 2; side-chains of residues within 4 Å of **NV-5138** are highlighted.

Recombinant human Sestrin2 with bound leucine was used to generate a crystal structure that was consistent with a previously published structure^12^ to aid in the design of specific ligands based on the binding of leucine. The free amino and carboxyl groups of leucine make extensive hydrogen bonds and salt bridge interactions with Glu451 and Arg390 residues respectively, while the side chain rests in a hydrophobic pocket lined by Leu389, Trp444, and Phe447. We hypothesized that larger side-chains, especially those with branching at the γ-carbon could form enhanced hydrophobic and van der Waals interactions within the lipophilic region of the leucine binding site. Accordingly, we synthesized novel binding ligands incorporating these structural features. These compounds were tested for Sestrin2 binding using a thermal shift assay and for their ability to activate mTORC1 in leucine-starved Human Embryonic Kidney (HEK)-293T cells. These studies led to the identification of **NV-5138** – a novel small molecule activator of mTORC1 signaling (Fig. 1a). **NV-5138** and leucine were shown to bind to Sestrin2 as evidenced by a dose-dependent positive shift in the melting temperature with increasing ligand concentration (Fig. 1b and Table 1). Further confirmation of binding by **NV-5138** and leucine was obtained by isothermal calorimetry (ITC) measurements, resulting in estimated Kd values of 1.49 µM and 1.55 µM, respectively (Fig. 1c, Supplementary Fig. 2a).

**Table 1:** The average shift +/− standard deviation in melting temperature (°C) of purified Sestrin2 in the presence of increasing concentrations of leucine or **NV-5138** (n = 3).

Compound Concentration (µM)	Average Tm shift (°C)	
	Leucine	NV-5138
1	0.61 ± 0.59	0.57 ± 0.22
10	3.64 ± 0.41	3.27 ± 0.16
100	6.49 ± 0.20	6.17 ± 0.18

The X-ray co-crystal structure of **NV-5138** in complex with Sestrin2 at 3.3 Å resolution was shown to be essentially identical to that of the leucine complex with no apparent conformational differences between the two structures (Fig. 1d). The binding pose of **NV-5138** is also largely conserved with the free amine and carboxyl groups making similar interactions as leucine and the side chain with the γ-difluoromethyl carbon making additional van der Waals interactions within the hydrophobic region of the leucine-binding pocket (Fig. 1e).

### NV-5138 modulates the Sestrin2-Gator2 interaction and activates mTORC1 in cells

**Figure 2:**
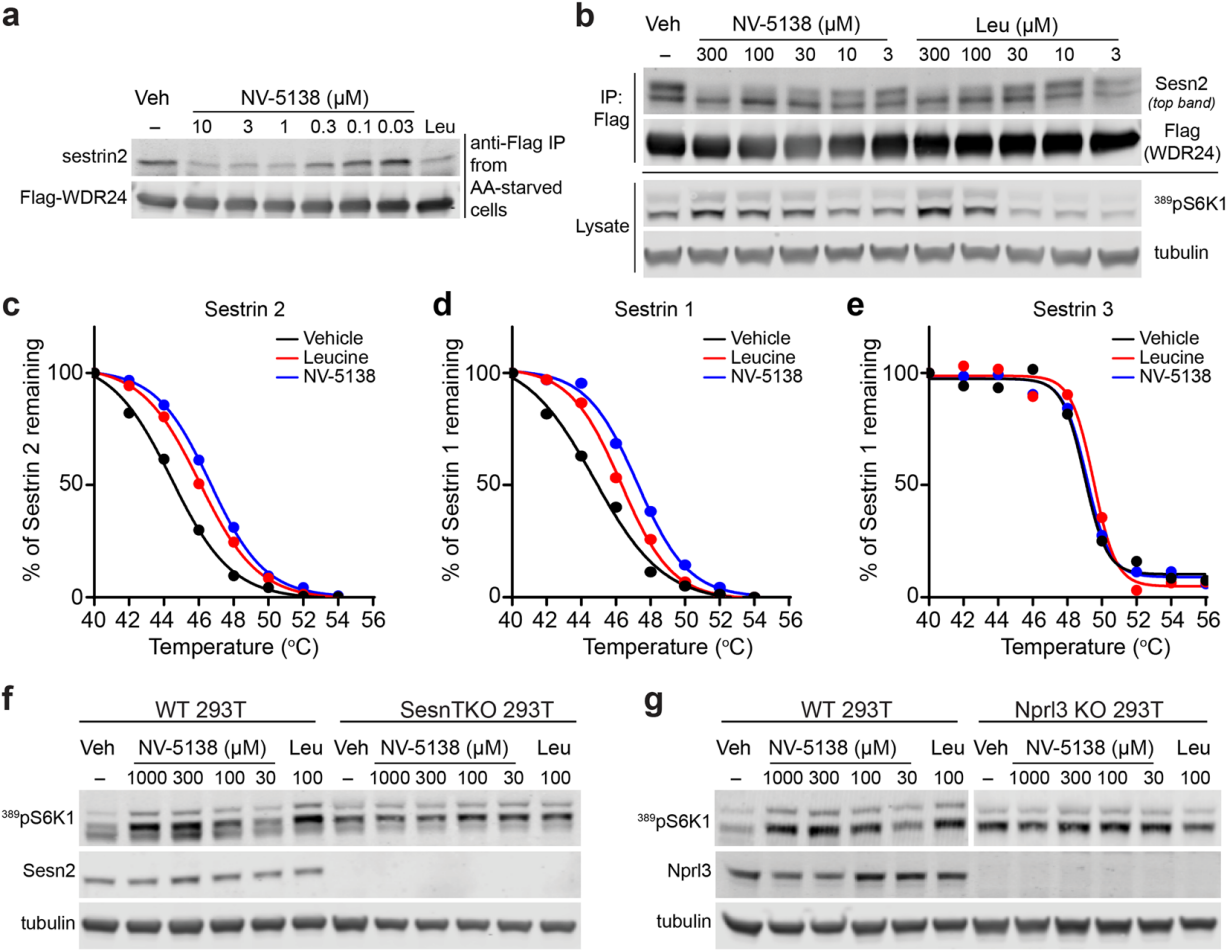
NV-5138 activates mTORC1 by modulating the interaction between Sestrin2 and GATOR2. (**a)** Immunoblotting of Sesn2 from the cell-free Sesn2/WDR24 interaction assay. Flag-WDR24 was immunoprecipitated from amino acid-starved Flag-WDR24 expressing HEK-293T cells, resuspended in cytosolic buffer and **NV-5138** or leucine (10 µM) was added for 10 min. (**b**) Dose-dependent activation of mTORC1 by **NV-5138** or leucine correlates with disruption of Sesn2 from Flag-WDR24. Flag-WDR24 expressing HEK-293T cells were starved of leucine for 50 min followed by addition of **NV-5138** or leucine for 10 min. Immunoblot shows levels of Sestrin2 bound to immunoprecipitated Flag-WDR24 (lower contrast images are available in Supplementary Fig. 6a) and levels of phosphorylated S6K1 (^389^pS6K1). (**c**) Cell based treatment with **NV-5138** shifts the melting temperature of intracellular Sestrin2 ~+2 °C. Percentage of Sestrin2 remaining compared to Sestrin2 amount at 40 °C is plotted as a function of temperature with non-linear regression performed to fit a melting curve. Values were determined from immunoblots of Sestrin2. (**d**) Cell based treatment with **NV-5138** shifts the melting temperature of intracellular Sestrin1 ~+ 2 °C. Curve generated as described in **(c)**. (**e**) Cell based treatment with **NV-5138** does not impact the melting temperature of intracellular Sestrin3. Curve generated as described in **(c)**. (**f**,**g**) Immunoblots of phosphorylated S6K1 (^389^pS6K1) to measure mTORC1 activity in unedited HEK-293T cells, HEK-293T cells deficient for sestrins 1,2 and 3 or HEK-293T cells deficient for the GATOR1 component Nprl3. Cells were starved for Leucine for 50 min followed by addition of **NV-5138** or leucine for 10 min. In (**g**) phosphorylated S6K1 (Thr389) samples have been run on two separate gels, original images are available in Supplementary Fig. 6b.

Leucine signals to mTORC1 by modulating the Sestrin2/GATOR2 interaction. Under leucine-depleted conditions, Sestrin2 is bound to GATOR2 and addition of leucine can disrupt this interaction within minutes leading to activation of mTORC1^10^. Given that **NV-5138** binds to Sestrin2 in a manner similar to leucine in a purified system, we measured the modulation of the Sestrin2/GATOR2 protein-protein interaction in response to **NV-5138** in a cell-free system and within intact cells using HEK-293T cells stably expressing flag-WDR24- a component of GATOR2^10^. Expression of flag-WDR24 allows for immunoprecipitation of the Sestrin2/GATOR2 complex from amino acid-starved cells with an anti-flag antibody. Similar to leucine, addition of **NV-5138** to immunoprecipitated Sestrin2/GATOR2 complexes rapidly disrupts the Sestrin2/GATOR2 interaction in a dose-dependent manner (Fig. 2a). Repeating the study in intact cells, addition of **NV-5138** to leucine-starved flag-WDR24 expressing 293 T cells prior to anti-flag immunoprecipitation of GATOR2 also resulted in disruption of GATOR2-bound Sestrin2 at doses correlating with activation of mTORC1 as evidenced by increases in phosphorylation of its downstream substrate S6K1 (^Thr389^pS6K1) (Fig. 2b) without affecting S6K1 expression (Supplementary Fig. 2b). To confirm that **NV-5138** is directly engaging Sestrin2 in cells, intracellular thermal shift analysis (CETSA) was performed by heating leucine-starved HEK-293T cells treated with **NV-5138** or leucine at a range of temperatures prior to processing for immunoblotting (Supplementary Fig. 2c)^23^. Plotting the percentage of Sestrin2 remaining as a function of temperature reveals addition of **NV-5138** or leucine increased the melting temperature of Sestrin2 by approximately 2 °C indicative of binding (Fig. 2c). We also used CETSA to measure the interaction of **NV-5138** and leucine with Sestrin1, which has been reported to bind leucine at similar potencies to Sestrin2, and Sestrin3 which reportedly does not bind leucine^24^. Similar to Sestrin2, **NV-5138** and leucine treatment also increased the melting temperature of Sestrin1 by approximately 2 °C (Fig. 2d). In contrast, neither **NV-5138** nor leucine changed the melting temperature of Sestrin3 (Fig. 2e). Taken together, **NV-5138** appears to stabilize Sestrin1 and Sestrin2, but not Sestrin3- consistent with previously published data for leucine binding. Finally, to determine whether Sestrins and the GATOR pathway are required for **NV-5138** activity, HEK-293T cells null for all three Sestrin isoforms or the GATOR1 component Nprl3 were obtained^10^. Treatment with **NV-5138** activates mTORC1 in leucine-starved unedited HEK-293T cells, but not in cells lacking all three Sestrins or the GATOR1 component Nprl3 indicating that compound activity requires an intact Sestrins/GATOR pathway (Fig. 2f,g).

### NV-5138 lacks proteinogenic capacity

Since **NV-5138** is an amino acid, we performed two different experiments to rule out any potential liabilities that could arise from the incorporation of **NV-5138** into proteins. We first determined whether **NV-5138** substitutes for any branched-chain amino acid or methionine into newly synthesized proteins using liquid chromatography followed by tandem mass spectroscopy (LC-MS/MS). **NV-5138** (100 µM) or vehicle was incubated with serum and leucine-starved 293T cells for 6 hours followed by proteomic analysis by mass spectrometry. Analysis of over 5000 tryptic peptides from vehicle or **NV-5138** treated cells showed no substitution of **NV-5138** for leucine, isoleucine, valine or methionine that would be evident by a change in mass (Supplementary Table 1). In contrast, addition of the methionine analog L-azidohomoalaine led to a large number of substitutions for methionine indicating that the analysis method was highly sensitive (Supplementary Table 1). To further test leucine incorporation into nascent proteins in the presence of **NV-5138**, a ^14^C-leucine incorporation assay in HeLa cells was used as previously described^25^. As expected, inhibition of protein synthesis with cyclohexamide or competition with non-radiolabeled leucine blocked incorporation of ^14^C-leucine into precipitated proteins (Supplementary Fig. 2d). In contrast, co-incubation with non-radiolabeled **NV-5138** did not show any competition against incorporation of ^14^C-leucine into precipitated proteins. High doses of **NV-5138** appeared to increase ^14^C-leucine incorporation consistent with increased translation mediated by increased mTORC1 pathway activation (Supplementary Fig. 2e).

### NV-5138 is not metabolized by Branched-Chain Amino Transaminase (BCAT)

BCAT is the primary enzyme that metabolizes leucine to alpha-ketoisocaproate (KIC). BCAT exists in two isoforms, mitochondrial BCAT2 which is ubiquitously expressed, and cytosolic BCAT1, which is predominantly expressed in embryonic tissues and the adult brain. To determine whether **NV-5138** is a substrate for BCAT isoforms, we established an *in vitro* enzymatic assay using purified BCAT1 and BCAT2 and performed the assay in the forward direction as described^26^. Transamination of L-leucine with alpha-ketoglutarate results in formation of alpha-ketoisocaproate, which is reductively aminated back to L-leucine by leucine dehydrogenase in the presence of ammonia and NADH. The disappearance of absorbance at 340 nm due to NADH oxidation is measured continuously over time. As expected, addition of leucine (0.015 to 1.5 mM), but not arginine, to the assay resulted in oxidation of NADH in a dose dependent manner indicating it was indeed transaminated by BCAT1 and BCAT2 (Supplementary Fig. 3a). However, we did not observe any transamination of **NV-5138** by BCAT1 or BCAT2 at any of the concentrations tested suggesting that **NV-5138** is resistant to this primary route of leucine metabolism (Supplementary Fig. 3b).

### NV-5138 is selective for Sestrin1/2

While mTORC1 activation by **NV-5138** appears dependent upon an intact Sestrins/GATOR2 pathway and not upon protein incorporation or transamination by BCAT, **NV-5138** was tested for off-target activity in the Eurofins/Cerep panel of over 100 potential targets. The selectivity panel included AMPA, kainate and NMDA receptor isoforms (Supplementary Table 2a–c). **NV-5138** showed no activity against any target up to 300 µM final concentration (highest concentration evaluated). **NV-5138** was further tested for the ability to functionally inhibit the activity of maximally-activated NMDA receptors expressed in *Xenopus laevis* oocytes by two electrode voltage-clamp electrophysiology^27^. As observed in the Eurofins/Cerep panel, there was no effect of **NV-5138** at 300 µM final concentration (data not shown). Therefore, **NV-5138** is a selective small molecule that binds to Sestrin1/2 and differentiates from the natural ligand leucine by the lack of metabolism via the BCAT pathway and utilization in protein synthesis.

### Pharmacokinetics (PK) and Pharmacodynamics (PD) Following Oral Administration of NV-5138

**Figure 3:**
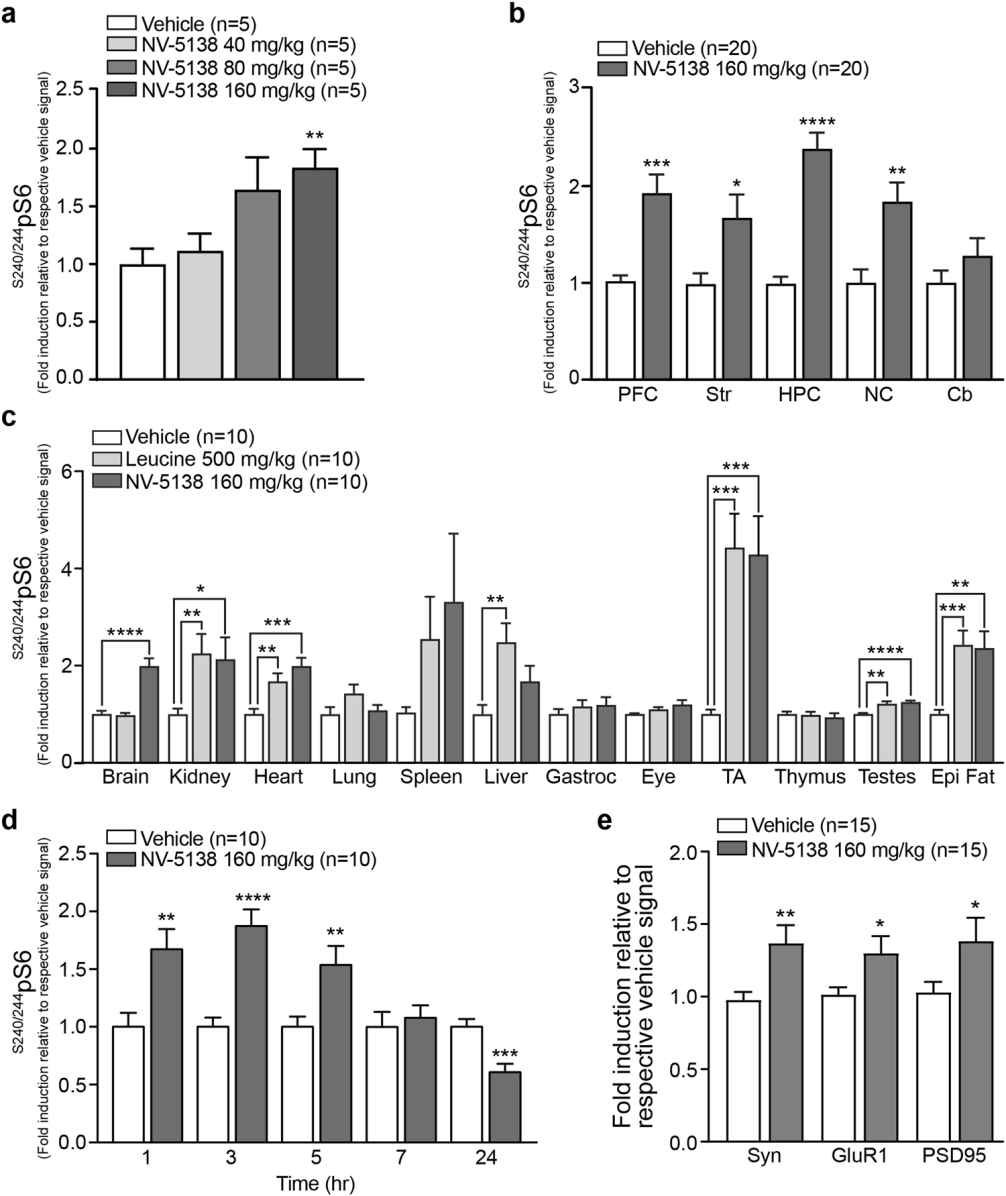
NV-5138 transiently activates mTORC1 in the brain. (**a**) Quantification of immunoblots for phosphorylated S6 (S240/44) from homogenized whole brains isolated from SD rats 1 h after oral dosing with **NV-5138** at 40 mg/kg, 80 mg/kg or 160 mg/kg (n = 5). (**b**) Quantification of immunoblots for phosphorylated S6 (S240/44) from synaptoneursomes from specific brain regions after dosing with vehicle or **NV-5138** (160 mg/kg, PO) for 1 h (n = 20). (**c**) Quantification of immunoblots for phosphorylated S6 (S240/44) from homogenized tissues from *ad libitum* fed SD rats orally dosed with leucine (500 mg/kg) or **NV-5138** (160 mg/kg) and sacrificed 1 h later (n = 10). (**d**) Quantification of immunoblots for phosphorylated S6 (S240/44) from homogenized whole brains isolated from SD rats 1, 3, 5, 7 and 24 h after oral dosing with **NV-5138** (160 mg/kg) (n = 10). (**e**) Quantification of immunoblots for GluR1, PSD95 and Synapsin 1 from homogenized synaptoneurosomes from specific brain regions collected from SD rats 24 h after dosing with vehicle or **NV-5138** (160 mg/kg, PO, n = 10). All data are mean ± SEM. *p < 0.05, **p < 0.01, ***p < 0.001 and ****p < 0.0001 indicates a significant difference by an unpaired two-tailed students t-test.

**NV-5138** was found to be essentially 100% orally bioavailable with an elimination half-life in plasma of ~ 3 h determined following intravenous and oral dosing in rats (Supplementary Table 3 and Fig. 4a). Given the favorable PK properties of **NV-5138**, we wished to determine whether oral administration of **NV-5138** could activate mTORC1 in the brain and other organs of *ad libitum* fed rats using the mTORC1 downstream substrate phosphorylated S6 (^S240/244^pS6) as a surrogate PD parameter for the activation status of the complex. As shown in Fig. 3a and Supplementary Fig. 5a, there was a dose-dependent increase in ^S240/244^pS6 in synaptoneurosomes isolated from the brain pre-frontal cortex (PFC) 1 h following dosing with a significant increase of ~ 2-fold versus vehicle observed at 160 mg/kg and a trending increase at 80 mg/kg. Based on this result we decided to perform subsequent experiments using the 160 mg/kg oral dose which resulted in a similar PK profile in plasma and whole brain extracts suggesting a high level of brain exposure (Supplementary Fig. 4b). One hour following a single oral administration of **NV-5138**, significant activation of mTORC1 across multiple brain regions in addition to the pre-frontal cortex including the striatum, hippocampus, neocortex but not the cerebellum was observed (Fig. 3b and Supplementary Fig. 5b).

In addition to the brain, we wanted to understand the effects of a single oral administration of **NV-5138** on selected peripheral organs compared to a single high dose of leucine in *ad libitum* fed rats. As shown in Fig. 3c and Supplementary Fig. 5c, there was significant activation of mTORC1 in kidney, heart, tibialis anterior muscle, testes, and epididymal fat. There was little to no activation observed in the lung, spleen, gastrocnemius muscle, eye or thymus while in the liver, there was significant activation seen following leucine administration and a trend with **NV-5138**. Thus, the extent of mTORC1 activation following **NV-5138** administration was similar to supplemental leucine across the selected peripheral tissues studied with the clear exception of the brain where there was no activation observed following leucine versus the ~2-fold increase following **NV-5138** administration.

We investigated the activation of mTORC1 in the brain further by determining the time course of mTORC1 activation following a single administration of **NV-5138**. As shown in Fig. 3d and Supplementary Fig. 5d), significant activation of mTORC1 peaked at ~3 h and was back to baseline/vehicle levels by 7 h which correlated with compound exposure shown in Supplementary Fig. 4b. The decrease in mTORC1 signaling in the **NV-5138** treated group 24 h following administration while significant was not consistently observed. Previous studies have demonstrated that transient activation of mTORC1 in the brain by agents such as ketamine, rapastinel and scopolamine result in increased translation of synaptic proteins which correlates to increased dendritic spine and synapse formation^16^. As shown in Fig. 3e and Supplementary Fig. 5e, there was a significant increase in the synaptic proteins Synapsin1, GluR1 and PSD95 observed 24 h following the single oral administration of **NV-5138** in synaptoneurosomes derived from the prefrontal cortex compared to vehicle treated animals. It should be noted that while the activation of mTORC1 in the prefrontal cortex correlated with compound exposure, the increased expression of synaptic proteins was observed well after compound levels have dropped and mTORC1 activity was back to baseline/vehicle levels. These data suggest a dissociation between compound exposure and pharmacodynamic efficacy as measured by the expression of several synaptic proteins which is similar to those agents that have been shown to require mTORC1 activation for improved synaptic plasticity and behavior in models of stress-induced depressive behavior^28^.

## Discussion

Competing mechanisms have been proposed to rationalize leucine sensing upstream of mTORC1^13,29^. The crystallographic and biochemical data we generated is consistent with Sestrin2 as a leucine-sensor upstream of mTORC1. The **NV-5138** binding mode to Sestrin2 is consistent with the leucine binding mode and subsequent activation of mTORC1 is correlated with disruption of Sestrin2 binding with GATOR2. Furthermore, CETSA confirms that both leucine and **NV-5138** increase the stability of Sestrin1 and Sestrin2 but not Sestrin3. These results are consistent with previously published reports that Sestrin3 only weakly binds leucine in contrast to Sestrin1 and 2. Finally, we have confirmed that mTORC1 signaling in mammalian cells null for all sestrins or for a component of GATOR1 is refractory to leucine as well as to **NV-5138**. Taken together, our data is consistent with **NV-5138** directly binding to Sestrin2 which facilitates its disassociation from GATOR2 and subsequently activates mTORC1.

Multiple studies have demonstrated robust activation of mTORC1 in peripheral tissues after oral administration of leucine and subsequent functional effects such as increased muscle protein synthesis and improved glucose homeostasis^19^. Similarly, **NV-5138** activates mTORC1 in multiple peripheral tissues similar to what has been reported for oral leucine administration including activation of mTORC1 in the heart, tibialis anterior and adipose tissue. One interesting exception was the gastrocnemius muscle, which has been previously shown to exhibit leucine sensitive mTORC1 signaling^30^. The lack of mTORC1 activation by leucine in the gastrocnemius in our study may be due to the fed state of the animals in contrast to previous studies that dose fasted animals. However, in a surprising contrast to leucine, **NV-5138** significantly activates mTORC1 in the brain following oral administration. While there are multiple conflicting studies of whether dietary ingestion of leucine or leucine-enriched diets activates mTORC1 in the hypothalamus or other regions of the brain^19,31^, our method of performing immunoblot analyses from homogenates generated from whole or micro-dissected brain after oral administration of a leucine bolus were unable to determine any activation of mTORC1. There are several key differences between **NV-5138** and leucine that may explain the differential activity of **NV-5138** in the brain. First and foremost, cerebrospinal fluid (CSF) levels of leucine are only a fraction (5–10%) of plasma concentrations^32,33^. The significantly lower CSF concentrations of leucine is accomplished by active sodium-dependent export of leucine from the brain to the plasma^34^. In contrast, the brain and plasma concentrations of **NV-5138** are equal and kinetics of clearance of **NV-5138** are similar between brain and plasma after oral administration. More work is required to determine whether this pharmacokinetic (PK) profile may be due to **NV-5138** not being a substrate of active sodium-dependent export from the brain. Second, radiolabeled tracer studies indicate that 80–90% of leucine transported into the brain incorporates into nascent protein or is transaminated by BCAT1 35 minutes after bolus administration^18^. Based on a suite of *in vitro* assays, **NV-5138** shows no evidence of being incorporated into protein or transaminated by BCAT1, which may further explain its brain PK profile and differential activation of mTORC1 in the brain versus leucine. Given the importance of leucine as the primary nitrogen donor for the biosynthesis of glutamate and gamma-aminobutyric acid (GABA) in the brain via transamination, the robust buffering of leucine levels in the brain may be a mechanism by which to regulate levels of these excitatory neurotransmitters^35^ providing a unique opportunity for the use of **NV-5138** as stable ligand for sestrin-mediated mTORC1 activation in the brain.

The mTORC1 pathway responds to multiple CNS-specific stimuli including growth factors such as BDNF, guidance molecules such as EphrinA1, and neurotransmitters such as glutamate^36^. In response to this wide range of stimuli, mTORC1 signaling has been implicated in a range of neural functions and neurological conditions including autism, epilepsy, memory formation, feeding behavior and mood disorders^37–40^. Control of protein synthesis and translation of synaptic proteins in dendrites by mTORC1 is thought to be an important mechanism for altering synapse structure and function^41^. Modulation of a number of receptor systems that can produce synaptic plasticity, including TrkB, NMDA, Group I mGluRs, and DRs have been shown to activate the mTORC1 pathway and are implicated in a wide range of neurobiological processes^36^. The magnitude of mTORC1 activation in the brain by **NV-5138** appears sufficient to increase levels of synaptic proteins 24 h after a single dose. Based on this initial observation, we plan on examining multiple functional outputs downstream of mTORC1 activation such as increased synaptogenesis and synaptic plasticity (R. Duman submitted). If confirmed, these functional changes due to activation of mTORC1 by **NV-5138** administration could impact therapeutic areas such as major depressive disorder and defects in learning and memory.

In summary, we have identified a novel, highly selective orally bioavailable amino acid that activates mTORC1 in the brain and selective peripheral organs. The identification of **NV-5138** also validates Sestrin1/2 as a new therapeutic target for the modulation of mTORC1 activity via an upstream regulatory mechanism. To our knowledge, this is the first selective mTORC1 activator targeting an amino acid sensor that is brain penetrant and achieves significant mTORC1 activation.

## Methods

### Protein production

Full-length, codon-optimized human Sestrin2 was cloned into a pMAL6H -C5XT bacterial expression vector. Protein was purified onto a HisTrap FF crude column (GE Healthcare) followed by anion exchange chromatography then further purified via size-exclusion chromatography. Further details can be found in Supplementary Information.

### Isothermal titration calorimetry (ITC)

Because **NV-5138** is an analog of the natural ligand L-leucine, and because it was known that leucine appears to co-purify with Sestrin2^12^, a procedure was developed to displace the endogenous leucine from the binding site and replace it with the weaker Sestrin2 ligand L-Methionine^24^. Methionine was later displaced by leucine and analogs for direct binding measurements. Details of **NV-5138** synthesis and protein preparation for ITC can be found in Supplementary Information.

### Crystallization and data collection

Optimized co-crystals were obtained from a 1 µl + 1 µl hanging drop using a reservoir solution of 1.35 M Na malonate pH 6.5 with 0.1 M MES pH 6.0, and a protein solution containing 200 µM **NV-5138**. Crystals were cryoprotected with 1.8 M Na Malonate pH 6.5 + 20% glycerol added directly to the drop, mounted in a cryoloop, and flash frozen in liquid nitrogen. Diffraction data at 3.0 Å were collected at the Canadian Light Source (CLS) beamline 08-ID1 and processed with XDS. Crystals belongs to space group I23 with 5 protein copies per asymmetric unit.

### Structure solution and refinement

The structure of the complex was solved by molecular replacement (MR) using the 5DJ4.pdb coordinates, without the solvent and ligand. The electron density maps are of high quality and confirm the previously published structures of Sestrin2. After multiple refinement cycles, the X-ray model was refined using data to 3.3 Å resolution and the final R factor is 18.4%. In order to confirm that the observed density was that of **NV-5138** and not of L-leucine, e-density maps were calculated using a model with leucine or **NV-5138** in the binding sites. All maps were contoured at the same levels of 1.5 rmsd for the 2Fo-Fc maps and 3.5 rmsd for the difference maps. Figures were prepared in PyMOL, diffraction data and refinement statistics for crystal structure of Sestrin2 bound to **NV-5138** are presented in Supplementary Table 4.

### Cell culture

All cell lines were cultured in DMEM supplemented with 10% FBS and maintained at 37 °C and 5% CO_2_. HEK-293T cells stably expressing Flag-WDR24 was generated as previously described^24^. For leucine starvation, cells were rinsed once with and incubated in leucine-free DMEM supplemented 10% dFBS for 50 min followed by treatment with **NV-5138**, leucine or vehicle for 10 min. Details of reagent used for cell culture can be found in Supplementary Information.

### Western blotting on immunoprecipitation (IP)

Cell lysates were prepared as previously described^24^. Lysates were cleared by centrifugation at 13,200 rpm at 4 °C for 8 min. For anti-FLAG IPs, the FLAG-M2 affinity gel was added to approximately 2 mg of lysates and incubated with rotation for 3 h at 4 °C. Following IP, the beads were washed one time with Triton wash buffer containing 500 mM NaCl. For lysate-based protein-protein interaction assay IPs were resuspended in cytosolic buffer containing vehicle, **NV-5138** or leucine at the indicated doses for 10 min. After compound incubation, IPs were collected by centrifugation. Immunoprecipitated proteins were denatured and, resolved by SDS-PAGE. Membranes were imaged using the LI-COR imaging system. Reagents and antibodies used are listed in Supplementary Information.

### Cellular thermal shift assay (CETSA)

CETSA was performed as previously described^23^. Briefly, HEK293T cells were incubated for an hour in the leucine-free media, then **NV-5138** (300 µM), leucine (300 µM) or vehicle (water) was added for an additional 30 min. Cells were then collected, centrifuged for 3 min at 300 *g* at room-temperature (RT), washed with PBS, centrifuged again, and finally resuspended in 1 ml of RT PBS containing EDTA-free protease-inhibitor tablets (Roche). 100 µl of each cell suspension was heated at the noted temperature for 3 min, samples were then placed on the bench at RT for 3 min before being flash-frozen in liquid nitrogen. To lyse the cells, samples were put through two cycles of freeze-thawing followed by centrifugation at 20,000 *g* for 20 min at 4 °C. 80 µl of the resulting supernatant was denatured and, resolved by SDS-PAGE. Membranes were imaged using the LI-COR imaging system. Antibodies used are listed in Supplementary Information.

### BCAT enzymatic assay

The assay buffer conditions were as follows: alpha-ketoglutarate (5 mM), NADH (0.075 mM), pyridoxal 5′-phosphate (5 µM), leucine dehydrogenase (0.95 U), ammonium sulfate (50 mM), DTT (5 mM) and vehicle (water), L-leucine, arginine, and **NV-5138** as indicated. The assay buffer was dispensed into a 96-well plate and brought up to 37 °C for 10 min before adding BCAT1 or BCAT2 (72 ng) in 0.1 M potassium phosphate buffer. Absorbance at 340 nm was then read every minute for 30 min at RT using an Envision plate reader. Source of each reagents used can be found in Supplementary Information.

### ^14^C-leucine protein incorporation assay

HeLa cells were washed once with PBS then incubated in starvation media (leucine and serum-free DMEM) containing CHX (10 ug/ml) or DMSO (1% final) for 2.5 h at 37 °C. After incubation, cells were washed once with PBS then incubated in labeling media containing ^14^C-leucine (2 µCi/ml) along with **NV-5138** or non-radioactive leucine for 30 min at 37 °C. After incubation, cells were harvested and lysed in Tris/HCl 10 mM pH 7.4 plus 1% NP40. Lysate were mixed with water and 20% trichloroacetic acid (1:1:2) and incubated on ice for 2 h to precipitate the protein. Proteins were collected by centrifugation, solubilized in NaOH, placed in scintillation fluid and read on a Wallac Microbeta Trilux 2450 with a 2-min count. Scintillation values were normalized as the percentage of the counts from the DMSO-treated wells.

### LC-MS/MS measurement of **NV-5138** protein incorporation

HEK-293T cells were washed once with PBS and incubated in leucine and serum-free DMEM for 1 h followed by addition of vehicle, **NV-5138** (100 µM) or AHA (100 µM) for 6 h. Cells were then lysed, and proteins were subjected to tryptic digestion after SDS-PAGE and analyzed by LC-MS/MS as previously described^42^. For the incorporation analysis, mass spectroscopy raw files were analyzed using the MaxQuant software package for peptide and protein identification. Details of the analysis can be found in Supplementary Information.

### Animals

Male Sprague–Dawley (SD) rats (Charles Research Laboratories) weighing 250–400 g were maintained in standard conditions with a 12-h light/dark cycle and ad libitum. Animals were randomly assigned to treatment groups for all experiments. All procedures were in accordance with IACUC and AAALAC guidelines. Experiments performed at Biomodels LLC were approved by Biomodels LLC′ IACUC (16-0614-2) and the Office of Laboratory Animal Welfare. Experiment performed at Chempartner were approved by AAALAC (license #00132) and Shanghai local license agency (SYXK(Shanghai)2017-0018).

### RNA *in situ* Hybridization Assay

RNA *in situ* hybridization for NeuN/Sesn1 and NeuN/Sesn2 mRNA was performed on automation using the RNAscope^®^ Reagent Kit (Advanced Cell Diagnostics, Inc., Newark, CA) according to the manufacturer’s instructions. Specific RNA staining signal was identified as red or turquoise, punctate dots. Samples were counterstained with Gill’s Hematoxylin. Brightfield images were acquired using an AperioAT2 digital slide scanner equipped with a 40X objective. Further details can be found in Supplementary Information.

### Western Blotting analysis of mTORC1 signaling and synaptic proteins

SD rats were dosed via oral gavage with **NV-5138**, leucine or vehicle (50 mg/ml in 0.5% MC and 0.1% Tween80 in water) at indicated doses and time points. After compound administration, animals were sacrificed by decapitation using a guillotine, immediately Specific regions of the brain were quickly microdissected, tissues collected, and flash frozen in liquid nitrogen. Peripheral tissues and all brain were homogenized using MP homogenizer in Lysis Buffer (Cell lysis buffer: 1% Triton X-100, 50 mM HEPES pH 7.4, 100 mM NaCl, 2 mM EDTA, 10 mM Beta-glycerophosphate, 10 mM Sodium pyrophosphate, and 1 protease inhibitor) at 4 °C. Synaptic proteins and mTORC1 analyses in each brain region were conducted on crude synaptoneurosome preparations as previously described^16^. Equal amounts of total protein from each sample were denatured and, resolved by SDS-PAGE. Membranes were imaged using the LI-COR imaging system. Proteins levels are normalized to tubulin or GAPDH levels and further normalized to vehicle treated rats for each tissue. Antibodies used are listed in Supplementary Information.

### Pharmacokinetic analysis of NV-5138 in rats

To determine oral bioavailability, male SD rats were dosed I.V at 1 mg/kg and PO at 5 mg/kg with **NV-5138** in 0.5% MC/0. 1% Tween80 (n = 3 per time point per group). Rats were fasted overnight for PO group and were given free access to food and water for IV group. After dosing, tail-vein blood was collected at the indicated time points into K2EDTA tubes and centrifuged at 2,000 *g* for 5 min to collect plasma. To measure levels of **NV-5138** in the brain and plasma, rats were dosed PO at 160 mg/kg (n = 5), sacrificed 1 h after dosing via decapitation and trunk blood and one hemisphere of the brain was collected. Plasma was combined with acetonitrile containing pregabalin as an internal standard and centrifuged, brain and heart samples were homogenized directly into acetonitrile. Levels of **NV-5138** were quantified via LC-MS/MS. WinNonlin V 6.2 statistics software (Pharsight Corporation, California, USA) was used to generate pharmacokinetics parameters using non-compartmental model.

### *In vitro* pharmacological screening

**NV-5138** was tested in duplicate at a concentration of 300 µM for potential interaction with a panel of 107 adverse drug effect targets including 21 transporters, receptors, ion channels and enzymes expressed in the CNS (Eurofins Pharma Discovery Services). The threshold to exclude a significant interaction was set as a binding/response of >50%.

### Quantification and statistical analysis

All statistical analyses were performed using Prism 7 (GraphPad Software). All data are presented as the means ± SD or ± SEM. The exact sample size (e.g., the number of mice or brains) of each experiment is provided in the relevant figures. Statistical analyses were conducted using t tests. All statistical comparisons were performed on data from biologically independent samples and replicated on different experimental days. Significance is shown as *p < 0.05, **p < 0.01, ***p < 0.001, ****p < 0.0001, and not significant values are not noted.

## Supplementary information

Dataset 1
