# Supplementary material for: Discovery of NV-5138, the first selective Brain mTORC1 activator
Source: Sci Rep. 2019 Mar 11;9:4107. doi: 10.1038/s41598-019-40693-5 (PMC6412019; doi:10.1038/s41598-019-40693-5)
Supplement: Supplementary file 1 — Dataset 1 [file 41598_2019_40693_MOESM1_ESM.docx]

Supplementary Information for

**Discovery of NV-5138, the first selective Brain mTORC1 activator**

Shomit Sengupta, Emilie Giaime, Sridhar Narayan, Seung Hahm, Jessica Howell, David O’Neill, George P. Vlasuk and Eddine Saiah

**This PDF file includes:**

Supplementary Methods

Supplementary Figures. 1 to 6

Supplementary Tables 1 to 4

References for Supplementary Information

**Supplementary Information Text**

**Supplementary Materials and Methods**

Reagents for cell culture, Western blotting (WB) and BCAT enzymatic assay

Reagents use for immunoprecipitation and WB are as follow: Triton lysis buffer (1% Triton, 10 mM β-glycerol phosphate, 10 mM pyrophosphate, 40 mM Hepes pH 7.4, 2.5 mM MgCl_2_ and 1 tablet of EDTA-free protease inhibitor (per 25 ml buffer)), cytosolic buffer (40 mM HEPES pH 7.4, 140 mM KCl, 10 mM NaCl, 2.5 mM MgCl2, 0.1% TritonX-100). Primary antibodies used for immunoprecipitation and WB were rabbit anti- T389 pS6K1 (Cell Signaling Technology; #9205), rabbit anti-S240/244 pS6 (Cell Signaling Technology; #5364), rabbit anti-GluR1 (Cell Signaling; #13185), rabbit anti-Synapsin 1 (Cell Signaling Technology; #5297), rabbit anti-PSD95 (Cell Signaling Technology; #9644), rabbit anti-GAPDH (Cell Signaling Technology; #5174), mouse anti-tubulin (Sigma-Aldrich; #T5168), rabbit anti-Sestrin2 (Cell Signaling Technology; # 8487), rabbit anti-Sestrin1 (Proteintech; #21668-1-AP), mouse anti-Sestrin2 (Sigma-Aldrich; # WH0083667M3), mouse anti-Sestrin3 (Sigma-Aldrich; # WH0143686M2) and anti-Flag M2 affinity gel (Sigma-Aldrich; # A2220). Secondary antibodies used for WB were goat anti-rabbit IRDye 800CW (#926-32211), donkey anti-mouse IRDye 680RD (926-68072) and goat anti-mouse IRdye680RD (#925-68070) from LI-COR

For cell culture, DMEM (Corning; #MT10013CV), Leucine-free DMEM (AthenasES; #0420), heat-inactivated FBS (Gibco; #16140-071) and dialyzed FBS (Gibco; #26400044).

Reagents used for BCAT enzymatic assay were: Recombinant protein of human BCAT1 (hBCAT1, OriGene; #TP319229), recombinant protein of human BCAT2 (hBCAT2, OriGene; #TP300625); L-leucine dehydrogenase from Bacillus cereus (Sigma-Aldrich; #L5135), pyridoxal 5’-phosphate hydrate (Sigma-Aldrich; #P3657), ammonium sulfate (Sigma-Aldrich; #A4418), alpha-ketoglutarate acid (Sigma-Aldrich; #75890), potassium phosphate dibasic (Sigma #P3786), potassium phosphate monobasic (Sigma-Aldrich; #795488), beta-nictotinamide adenine dinucleotide, NADH (Acros Organics; #271100010).

Protein production

Full-length, codon-optimized human Sestrin2 was cloned into a pMAL6H -C5XT bacterial expression vector such that Sestrin2 became N-terminally fused with a Tobacco Etch Virus nuclear-inclusion-a endopeptidase (TEV)–cleavable His6-MBP tag. This vector was transformed into Escherichia coli LOBSTR (DE3) cells (Kerafast) ^1^. Cells were grown at 37 °C to 0.6 OD, then protein production was induced with 0.2 mM IPTG at 18 °C for 12–14 h. Cells were collected by centrifugation at 6,000g, resuspended in lysis buffer (50 mM Tris-HCl, pH 8.5, 400 mM NaCl, 5 mM MgCl_2_, 1 mM dithiothreitol (DTT), 0.2% CHAPS, 10% glycerol and 1 tablet of protease inhibitor tablets per 50 ml (Complete Inhibitor tablets from Roche w/o EDTA)), homogenized and sonicated. The lysate was cleared by centrifugation at 15,000g for 45 min. The soluble fraction (~250 ml) was loaded onto a HisTrap FF crude column (GE Healthcare) at 2 ml/min followed by washing with lysis buffer at 4 ml/min. After washing, the protein was eluted in lysis buffer containing 300 mM imidazole at 4 ml/min. Fractions corresponding to the Sestrin2 protein were identified via SDS-PAGE and pooled. The eluted Sestrin2 was then incubated with TEV protease and dialyzed overnight at 4 °C into 50 mM Tris-HCl, pH 8.5, 400 mM NaCl, 5 mM MgCl_2_, 1 mM dithiothreitol (DTT), 0.1% CHAPS, 10% glycerol. To separate the N-terminal tag from the cleaved Sesrin2, from cleaved sample was then re-applied to the HisTrap FF crude column and flow-through collected. The protein was then subjected to anion exchange chromatography on an 8ml Mono Q column (GE Healthcare) with a linear NaCl gradient and then further purified via size-exclusion chromatography on a Superdex S200 26/60 column (GE Healthcare) equilibrated in running buffer (50 mM Hepes pH 7.4, 200 mM NaCl, 1 mM DTT, 2.5 mM MgCl_2_, and 5% glycerol).

Protein preparation for isothermal titration calorimetry

For this exchange procedure ion exchange chromatography was used using the following buffers:

Buffer-A: HEPES 20mM pH 7.4, βME 5mM, Buffer-B: HEPES 20mM pH 7.4, βME 5mM, Methionine 5mM, Buffer-C: HEPES 20mM pH 7.4, βME 5mM, NaCl 1M. Sestrin2 (6 mg) was diluted to 5ml with buffer-A, and loaded on a MonoQ HR 5/50 column equilibrated in buffer-A. The column was washed with 2-3 column volume (CV) of buffer-A followed by 130 ml of buffer-B. The protein was then eluted in steps of 300 mM, 500 mM, and 1 M NaCl in buffer-C. The protein eluted in the 300 mM NaCl fractions and was concentrated to 7.6 mg/ml.

LC-MS/MS measurement of **NV-5138** protein incorporation

Over 5000 tryptic peptides were analyzed for each compound treatment. For each peptide, potential **NV-5138** incorporation can be identified due to the specific mass difference of **NV-5138** compared to the masses of leucine, isoleucine, valine, and methionine. To generate a false-positive rate, peptides isolated from vehicle-treated cells were analyzed for a similar change in mass as would be expected with incorporation of **NV-5138**. Given the large structural and charge difference between **NV-5138** and lysine, lysine substitution was measured as a negative control. The substitution of methionine by the AHA was used as a positive control ^2^.

RNA *in situ* Hybridization Assay

Target probes for RNA in situ hybridization were designed for rat Sestrin1 (NM_001106396.1) by targeting nucleotides 695-1754; for rat Sestrin2 (NM_001109358.2) by targeting nucleotides 614-1595; for NeuN (NM_001134498.2) by targeting nucleotides 16-1116. Each probes were design by Advanced Cell Diagnostics, Inc and validated by an *In Silico* process to minimize cross-hybridization with the rest of the genome. Moreover, the double ZZ probe design increases the hybridization specificity which, combined with the high sensitivity obtained with the RNAscope^®^ method, results in a high signal-to-noise ratio. RNA in situ hybridization for NeuN/Sesn1 and NeuN/Sesn2 mRNA was performed on automation using the RNAscope^®^ Reagent Kit (Advanced Cell Diagnostics, Inc., Newark, CA). 5 μm formalin fixed, paraffin embedded (FFPE) coronal brain sections from two male Sprague Dawley rats were pretreated at 95ºC for 10 min followed by protease treatment for 15 min at 40ºC prior to hybridization with the target oligo probes. Preamplifier, amplifier, and HRP/AP-labeled oligos were then hybridized sequentially, followed by chromogenic precipitate development. Each sample was quality controlled for RNA integrity with an RNAscope^®^ probe specific to PPIB/POLR2A RNA and for background with a probe specific to bacterial dapBRNA (Supplementary Figure 1b). Specific RNA staining signal was identified as red or turquoise, punctate dots. Samples were counterstained with Gill’s Hematoxylin. Brightfield images were acquired using an AperioAT2 digital slide scanner equipped with a 40x objective.

Synthesis of **NV-5138**

*Synthesis of (S)-2-amino-5,5-difluoro-4,4-dimethylpentanoic acid:*

*Synthetic scheme:*

*Procedures and characterization:*

*Step 1: diethyl 2-(1,1,1-trifluoropropan-2-ylidene)malonate：*

To an ice-cold flask containing THF (1 L) was added TiCl_4_ (65.8 ml, 0.6 mmol) dropwise over 20 mins, CCl_4_ (30 ml) was then added followed by diethyl malonate (48.0 g, 0.3 mol) and 1,1-difluoropropan-2-one (56.4 g, 0.6 mmol). The mixture was warmed to room temperature and stirred for 18h then cooled to 0°C and pyridine (200 ml) was added dropwise over 20 mins then stirred at room temperature overnight. The reaction mixture was poured into water (3 L), filtered, and the filtrate was extracted with EtOAc (500 ml x 3). The combined organic layers were washed with water (600 ml), 1M HCl (600 mm x 2), water (600 ml), sat. NaHCO_3_ (600 ml) and brine (600 ml) then dried (Na_2_SO_4_), filtered and concentrated in vacuo. The resulting residue was purified by chromatography (silica gel, ethyl acetate/petroleum ether from 0% to 5%) to afford diethyl 2-(1,1-difluoropropan-2-ylidene)malonate (60.9 g, 258 mmol, 86%) as a colorless liquid.

ESI-MS (EI^+^, m/z): 237.0 [M+H]^+^.

^1^H-NMR (500 MHz, CDCl_3_): δ 6.97 (t, *J* = 55.5 Hz, 1H), 4.25-4.33 (m, 4H), 2.03 (s, 3H), 1.29-1.34 (m, 6H).

*Step 2: diethyl 2-(1,1-difluoro-2-methylpropan-2-yl)malonate:*

To a solution of diethyl 2-(1,1-difluoropropan-2-ylidene)malonate (40.0 g, 0.17 mol) and CuI (48.4 g, 0.25 mol) in DCM (400 ml) and THF (100 ml) was added MeMgI (113 ml, 3M in Diethyl ether, 0.34 mol) dropwise at -20 ^o^C over 1h. The solution was poured into ice-water (1 L) and treated with sat. NH_4_Cl solution (500 ml), the mixture was stirred for 30 mins and filtered, the filtrate was extracted with DCM (500 ml), the organic phase was washed with water (400 ml x 2), and brine (400 ml), dried (Na_2_SO_4_), filtered and concentrated in vacuum to afford diethyl 2-(1,1-difluoro-2-methylpropan-2-yl)malonate (40.4 g, 0.16 mol, 95%) as a brown liquid which was used for the next step.

ESI-MS (EI^+^, m/z): 253.1 [M+H]^+^.

^1^H-NMR (500 MHz, CDCl_3_): δ 5.94 (t, *J* = 57.5 Hz, 1H), 4.17-4.23 (m, 4H), 3.50 (s, 1H), 1.26-1.29 (m, 6H) , 1.20 (s, 6H).

*Step 3: 4,4-difluoro-3,3-dimethylbutanoic acid:*

To a solution of diethyl 2-(1,1-difluoro-2-methylpropan-2-yl)malonate (150.0 g, 0.6 mol) in DMSO (1.25 L) and H_2_O (125 ml) was added LiOH.H_2_O (125.0 g, 3 mol) and the resulting mixture heated to 90^o^C for 2 h. The reaction was then diluted with water (10 L) and extracted with DCM (2.5 L). The aqueous phase was adjusted to pH 3-4 with 6M HCl solution then extracted with DCM (1.5 L×2), dried (Na_2_SO_4_), filtered and concentrated in vacuo to afford 4,4-difluoro-3,3-dimethylbutanoic acid (101 g, crude) as a brown liquid.

ESI-MS (EI^+^, m/z): 151.1 [M-H]^-^.

^1^H-NMR (500 MHz, DMSO-*d*_6_): δ 12.30 (s, 1 H), 5.82 (t, *J* = 56.5 Hz, 1H), 2.26 (s, 2 H) , 1.03 (s, 6 H).

*Step 4: 4,4-difluoro-N-methoxy-N,3,3-trimethylbutanamide:*

To a solution of 4,4-difluoro-3,3-dimethylbutanoic acid (101 g, 0.63 mol), N,O-dimethylhydroxylamine hydrochloride (122.8 g, 1.26 mol) and HATU (359.1 g, 0.94 mol) in DMF (1 L) was added Et_3_N (318.2 g, 3.15 mol) at 0^o^C, and the reslting mixture stirred at rt for 17h. The reaction was filtered, and the filtrate was diluted with water (3 L) and extracted with Et_2_O (1 L×3). The combined organic layers were washed with 1M HCl (1 L×2), sat. NaHCO_3_ solution (1 L), water (1 L) and brine (1 L) then dried (Na_2_SO_4_), filtered and concentrated in vacuo to afford 4,4-difluoro-N-methoxy-N,3,3-trimethylbutanamide (100 g, 0.51 mol, 81%, 2 steps) as a brown liquid.

ESI-MS (EI^+^, m/z): 196.0 [M+H]^+^.

^1^H-NMR (500 MHz, CDCl_3_): δ 5.96 (t, *J* = 57.5 Hz, 1H), 3.69 (s, 3H), 3.17 (s, 3H), 2.51 (s, 2H) , 1.12 (s, 6H).

*Step 5:* *4,4-difluoro-3,3-dimethylbutanal:*

To a solution of 4,4-difluoro-N-methoxy-N,3,3-trimethylbutanamide (40.0 g, 0.20 mol) in THF (400 ml) at 0°Cwas added LiAlH_4_ (200 ml, 1 M in THF, 0.20 mol) dropwise. After stirring for 1h the mixture was quenched with citric acid solution (1 L) then extracted with Et_2_O (500 ml x 2). The combined organic layers were washed with brine (100 ml), dried (Na_2_SO_4_) and the resulting solution used without further purification in the next step.

*Step 6: 5,5-difluoro-4,4-dimethyl-2-((S)-1-phenylethylamino)pentanenitrile:*

To a crude solution of 4,4-difluoro-3,3-dimethylbutanal in diethyl ether (1 L) at 0^o^C was added (S)-1-phenylethanamine (40 ml), AcOH (40 ml) and TMSCN (40 ml) and the resulting solution allowed to warm to rt over 17h. The reaction mixture was diluted with ethyl acetate (500 ml), washed with H_2_O (500 ml x 2) and concentrated to afford 5,5-difluoro-4,4-dimethyl-2-((S)-1-phenylethylamino)pentanenitrile (40.5 g, crude) as a brown liquid.

ESI-MS (EI^+^, m/z): 267.0 [M+H]^+^.

*Step 7: (S)-5,5-difluoro-4,4-dimethyl-2-((S)-1-phenylethylamino)pentanoic acid:*

A solution of 5,5-difluoro-4,4-dimethyl-2-((S)-1-phenylethylamino)pentanenitrile (40.5 g, crude) in conc. HCl (500 ml) and AcOH (100 ml) was heated to 100^o^C for 64h then concentrated. The pH was adjusted to 12 with 1M NaOH solution then extracted with petroleum ether (200 ml). The aqueous phase was adjusted to pH 5-6 with 6M HCl and a white solid formed. This was filtered, and the resulting filter cake was washed with water (50 ml) and dried in vacuo to obtained 20.3 g white solid. This solid was purified by chiral prep-HPLC to afford (S)-5,5-difluoro-4,4-dimethyl-2-((S)-1-phenylethylamino)pentanoic acid (10.5 g, 0.037 mol, 18.5%) as a white solid.

ESI-MS (EI^+^, m/z): 286[M+H] ^+^, RT=1.23 min.

^1^H-NMR (400 MHz, DMSO-*d_6_*): δ 7.23-7.35 ( m, 5 H), 5.77 (t, *J* = 57.2 Hz, 1H), 3.60-3.66 (m, 1H), 2.77 (m, 1H), 1.50 (d, J=6.8 Hz,2 H)1.26 (d, *J* = 6.8 Hz, 3H), 0.78 (d, *J* = 9.2 Hz, 6H).

Chiral HPLC (Column: Cellulose-SC 4.6*100mm 5um) RT = 0.84 min, 100%.

*Step 8: (S)-2-amino-5,5-difluoro-4,4-dimethylpentanoic acid:*

To a solution of (S)-5,5-difluoro-4,4-dimethyl-2-((S)-1-phenylethylamino)pentanoic acid (20 g, 0.07 mol) in MeOH (200 ml) was added HCOONH_4_ (22.1 g, 0.35 mol) and Pd/C (10%, 2.0 g). The resulting reaction mixture was heated to 60^o^C for 4h then filtered, concentrated then diluted with water (50 ml) and filtered. The filtrate was concentrated, and the crude was purified via reverse phase chromatography to afford (S)-2-amino-5,5-difluoro-4,4-dimethylpentanoic acid (10 g, 0.055 mol, 78.9%) as a white solid.

ESI-MS (EI^+^, m/z): 182.0[M+H]^+^.

^1^H-NMR (500 MHz, DMSO-*d_6_*): δ 7.70 (s, 3 H), 5.79 (t, *J* = 56.5 Hz, 1H), 3.20(t, *J* = 6.0 Hz, 1H), 2.01 (dd, *J* = 14.5 Hz, *J* = 5.0 Hz, 1H), 1.49 (dd, *J* = 14.5 Hz, *J* = 7.0 Hz, 1H), 0.97 (d, *J* = 3.0 Hz, 6H).

Supplementary Figures


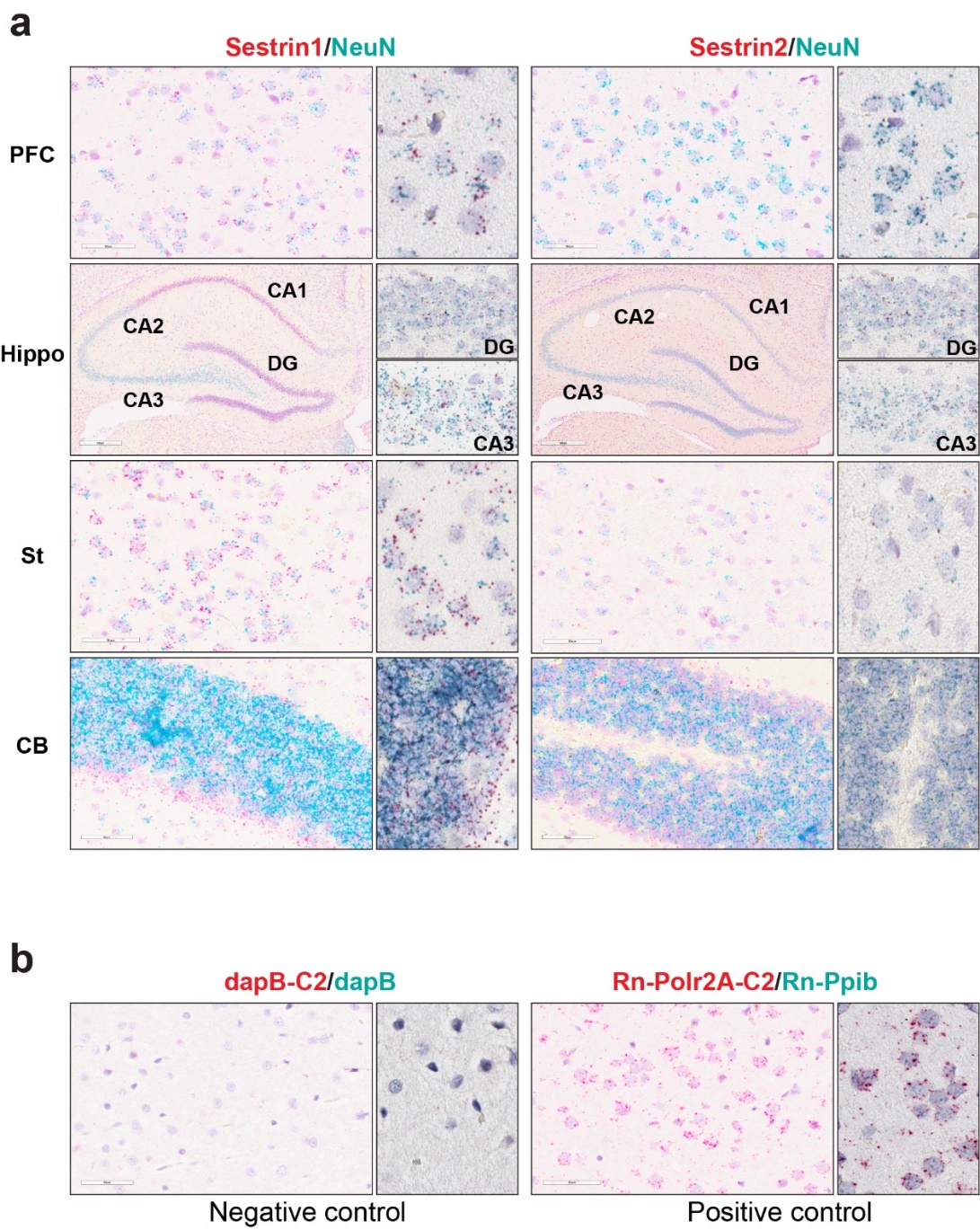


**Supplementary Figure 1.** **RNA *in situ* hybridization for Sestrin1 and Sestrin2.**

**a**. RNA *in situ* hybridization for Sestrin1 (red) and Sestrin2 (red) shows clear expression and co-localization with the neuronal marker NeuN (turquoise) in multiple regions of the brain. Representative images are shown for the prefrontal cortex (PFC), hippocampus (Hippo), striatum (St) and cerebellum (CB). Within the hippocampus, the dentate gyrus (DG) and cornu ammonis (CA) fields CA1, CA2 and CA3 are shown. **b**. Representative images of the positive control (using Rn-Ppib/Rn-Polr2a probe) and negative control (using dapB/dapB probe) use to assess the RNA quality.


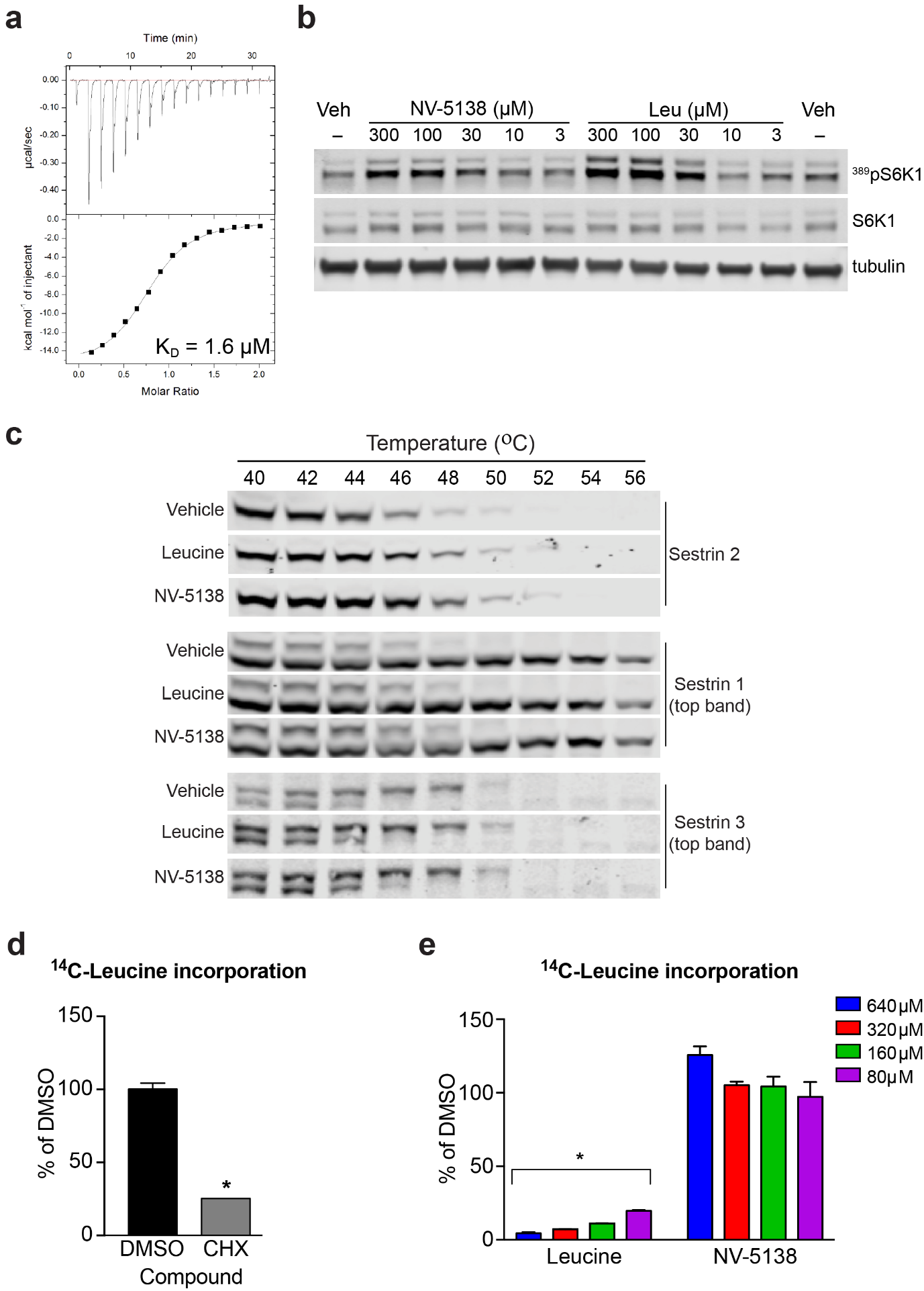


**Supplementary Figure 2. Leucine and NV-5138 bind Sestrin 2 with similar potency but NV-5138 is non-proteinogenic.**

**a.** Measurement of the binding affinity of leucine for Sestrin2 by isothermal calorimetry (ITC) predicts a binding K_d_ of 1.6 µM with a molar stoichiometry of 1. B. **b.** Dose-dependent activation of mTORC1 by **NV-5138** or Leucine. HEK-293T cells were starved of Leucine for 50 min followed by addition of **NV-5138** or Leucine for 10 min. Immunoblot shows levels of of phosphorylated S6K1 (^S389^pS6K1) and total S6K1. **c.** Immunoblots of Sestrin2, Sestrin1 (top band) and Sestrin3 (top band) from intracellular thermal shift studies described in Fig. 2C-E. **d.** Validation of assay measuring ^14^C-Leucine incorporation into nascent protein by cyclohexamide treatment in HeLa cells. **e.** Non-radiolabeled leucine competes with ^14^C-Leucine incorporation into precipitated proteins in HeLa cells while similar doses of **NV-5138** do not. Increase in ^14^C-Leucine incorporation at highest dose of **NV-5138** is statistically significant versus DMSO treated samples (p<0.05, student t-test).


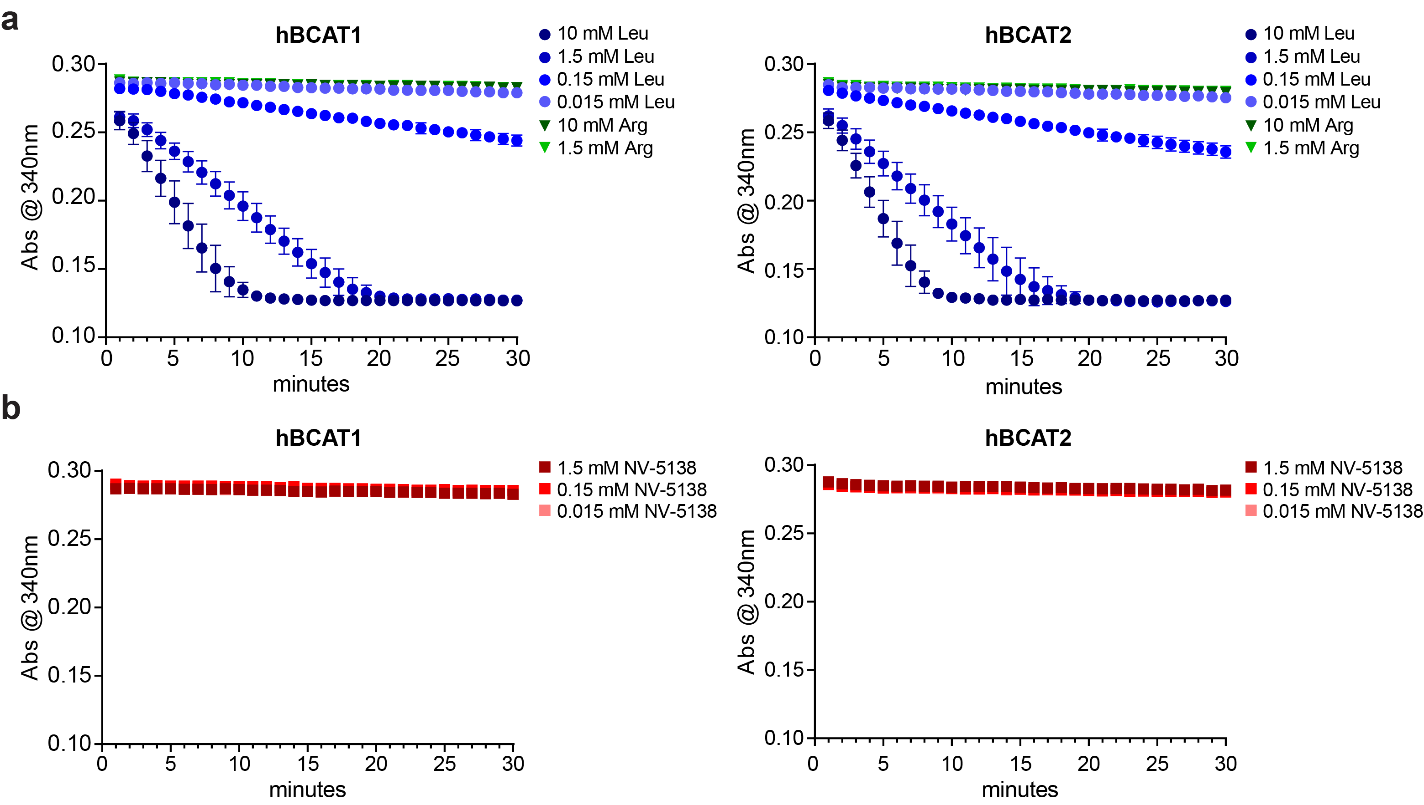


**Supplementary Figure 3. NV-5138 is not a substrate for BCAT1 or BCAT2.**

Transamination of L-leucine with alpha-ketoglutarate by BCAT1 or BCAT2 results in formation of alpha-ketoisocaproate, which is reductively aminated back to L-leucine by leucine dehydrogenase in the presence of ammonia and NADH. The disappearance of absorbance at 340 nm due to NADH oxidation is measured continuously over time. **a.** Leucine, but not arginine, shows a dose-dependent decrease in absorption at 340 nm indicative of transamination by BCAT1 and BCAT2. **b.** **NV-5138** shows no decrease in absorption at 340 nm when tested up to 1.5 mM. The data is represented as the mean ± SD n=3/time point


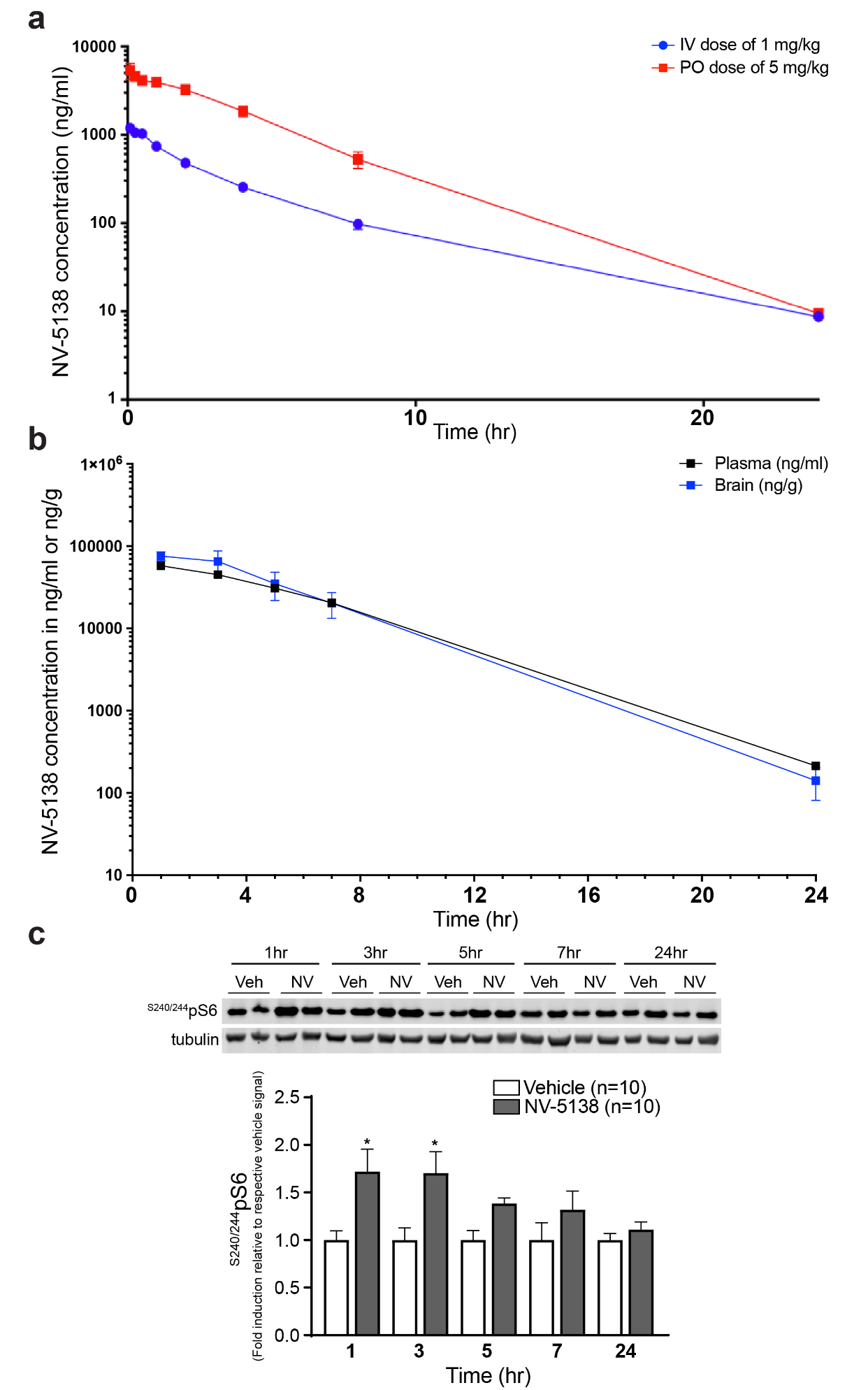


**Supplementary Figure 4. NV-5138 is 100% orally bioavailable.**

**a.** Mean plasma concentration-time profiles of **NV-5138** after an IV dose at 1 mg/kg (blue) and a PO (red) dose at 5 mg/kg in Male SD rat. The data is represented as the mean±SD n=3/time point. Pharmacokinetic analysis of IV/PO study using WinNonlin V 6.2 statistics software is presented in Supplementary Table 3. **b.** Mean plasma and brain concentration-time profiles of **NV-5138** after PO dosing at 160 mg/kg in male SD rats. The data is represented as the mean±SD n=3/time point. The compound exposure correlates with mTORC1 signaling data represented in Fig. 3d and Supplementary Figure 4C **c.** Quantification of immunoblots for phosphorylated S6^(S240/44)^ from homogenized hearts from SD rats 1, 3, 5, 7 and 24 hours after being orally dosed with **NV-5138** (160 mg/kg) (n=10). Phosphorylated S6 levels are normalized to tubulin levels and further normalized to vehicle treated rats for each tissue. Representative immunoblots for phosphorylated S6^(S240/44)^ from heart tissue from vehicle and **NV-5138** treated animals shown above. All data are mean ± SEM. *p<0.05 indicates a significant difference by an unpaired two-tailed students t-test.

**
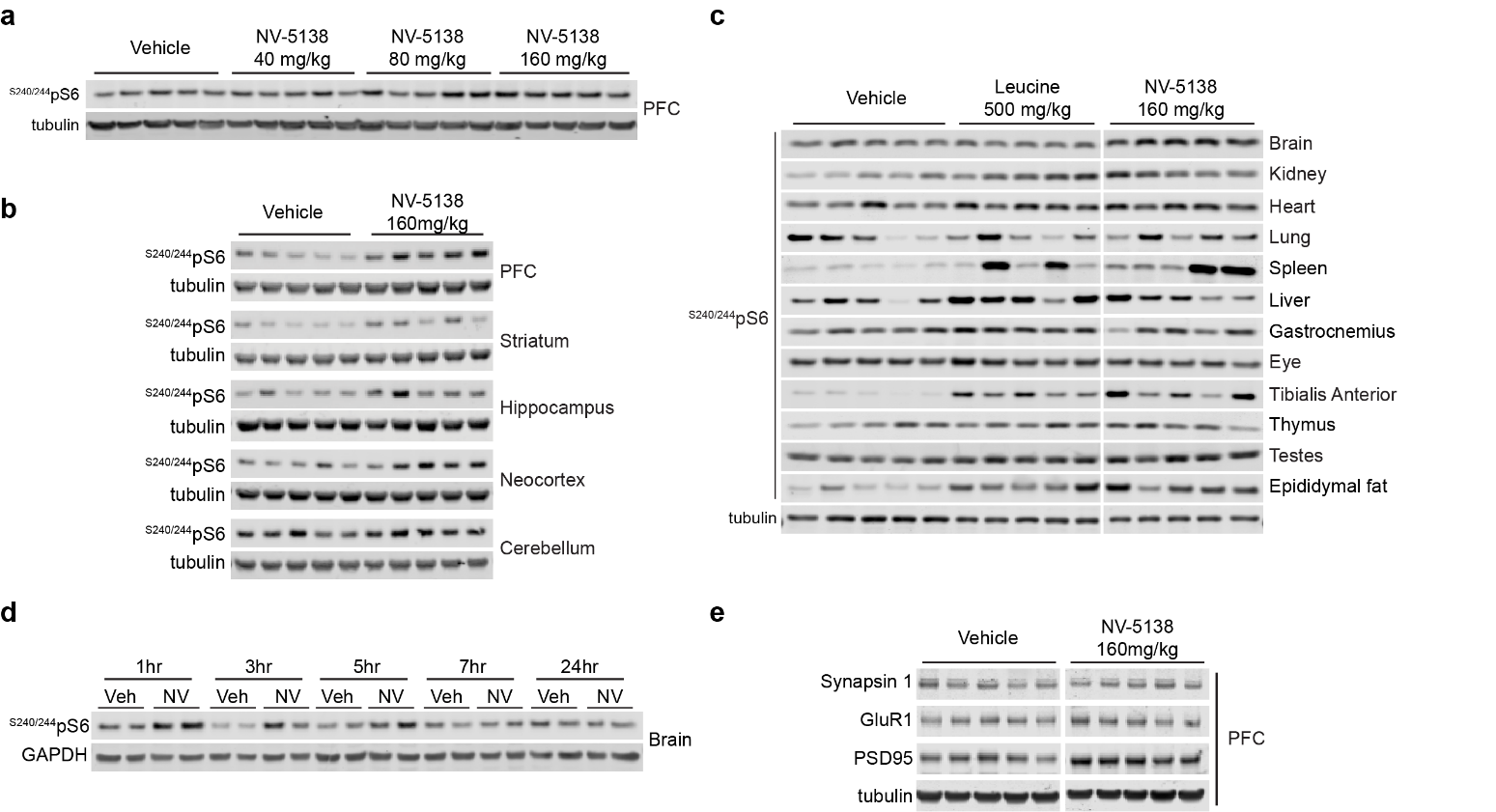
**

**Supplementary Figure 5. NV-5138 transiently activates mTORC1 in the brain.**

**a.** Representative immunoblots for normalized phosphorylated S6^(S240/44)^ data shown in Fig. 3a. **b.** Representative immunoblots for the normalized phosphorylated S6^(S240/44)^ data shown in Fig. 3b. **c.** Representative immunoblots for the normalized phosphorylated S6^(S240/44)^ data shown in Fig. 3c. **d.** Representative immunoblots for the normalized phosphorylated S6^(S240/44)^ data shown in Fig. 3d. **e.** Representative immunoblots for Synapsin 1, GluR1 and PSD95 as shown in Fig. 3e.


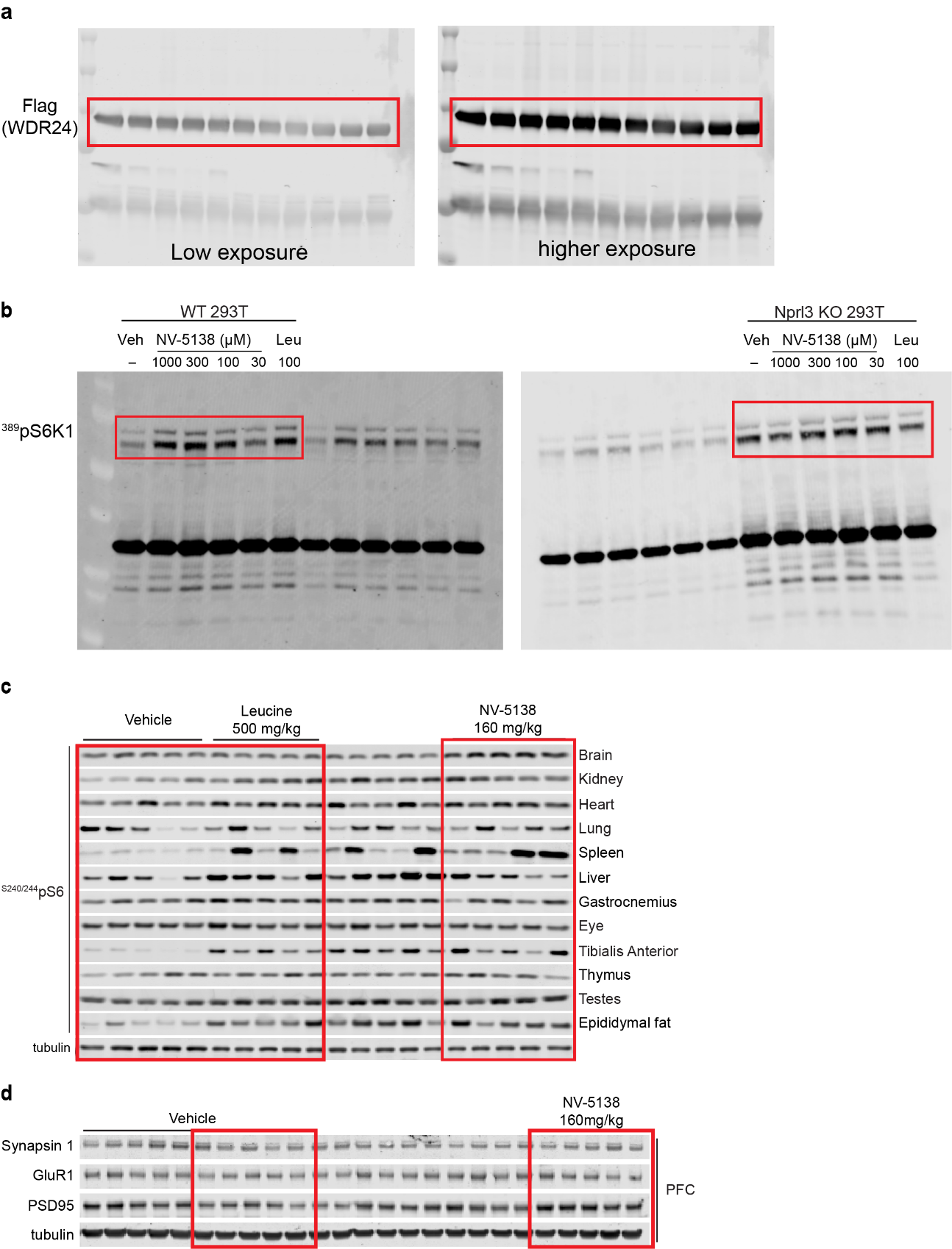


**Supplementary Figure 6. Original full-length western blot images.**

**a.** Blot showing two different exposure for WDR24 Flag expression for Fig. 2b. **b.** Original blot of Fig. 2g showing that phosphorylated S6K1^(389)^ blot originated from two different blot. **c.** Original blot of Supplementary Fig. 5c. **d**. Original blot of Supplementary Fig. 5e.

**Supplementary Tables**


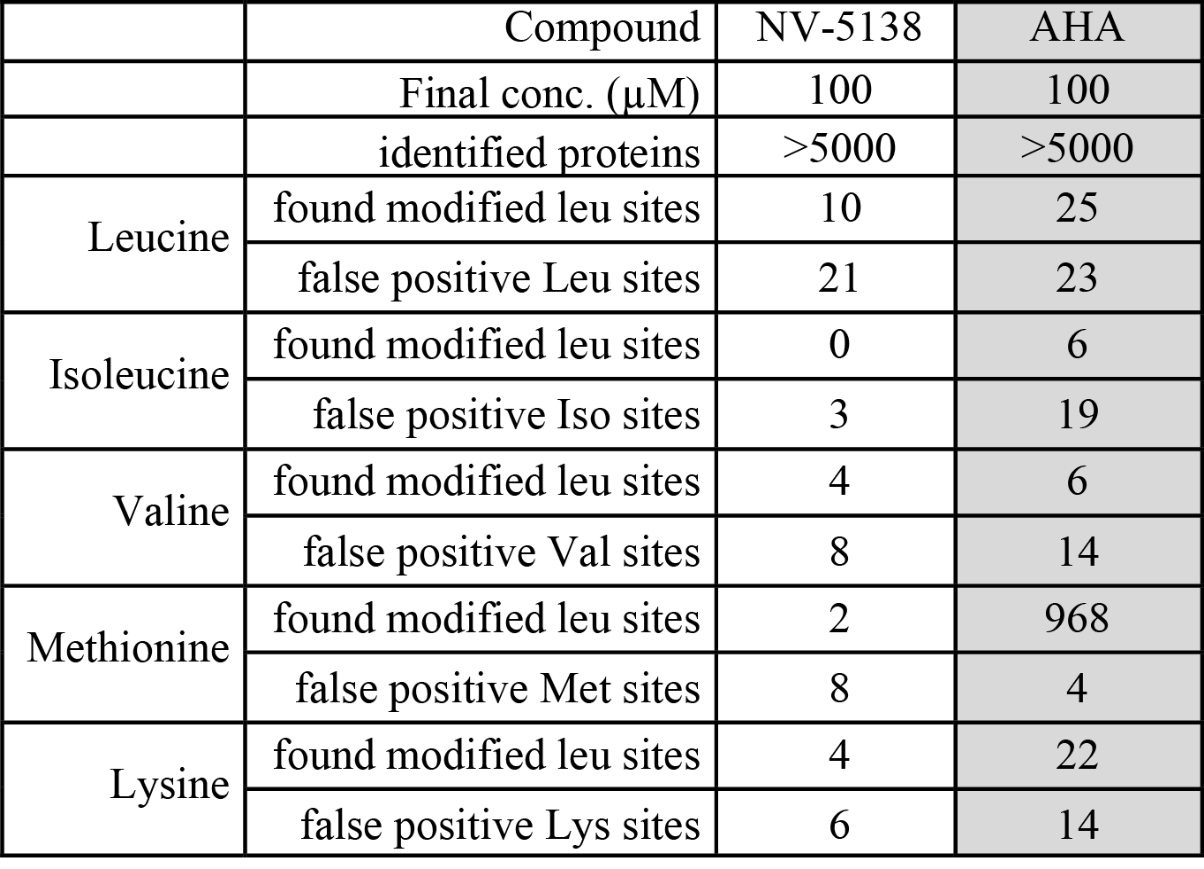


**Supplementary Table 1:** Number of tryptic peptides with mass changes consistent with substitution with **NV-5138** from leucine and serum starved 293T cells incubated with vehicle (listed as false positive), **NV-5138** (100µM) or L-azidohomoalaine (100µM, AHA) for 6 hours.

Supplementary Table 2. Selectivity panel

Supplemental Table 2A: CEREP Selectivity Panel


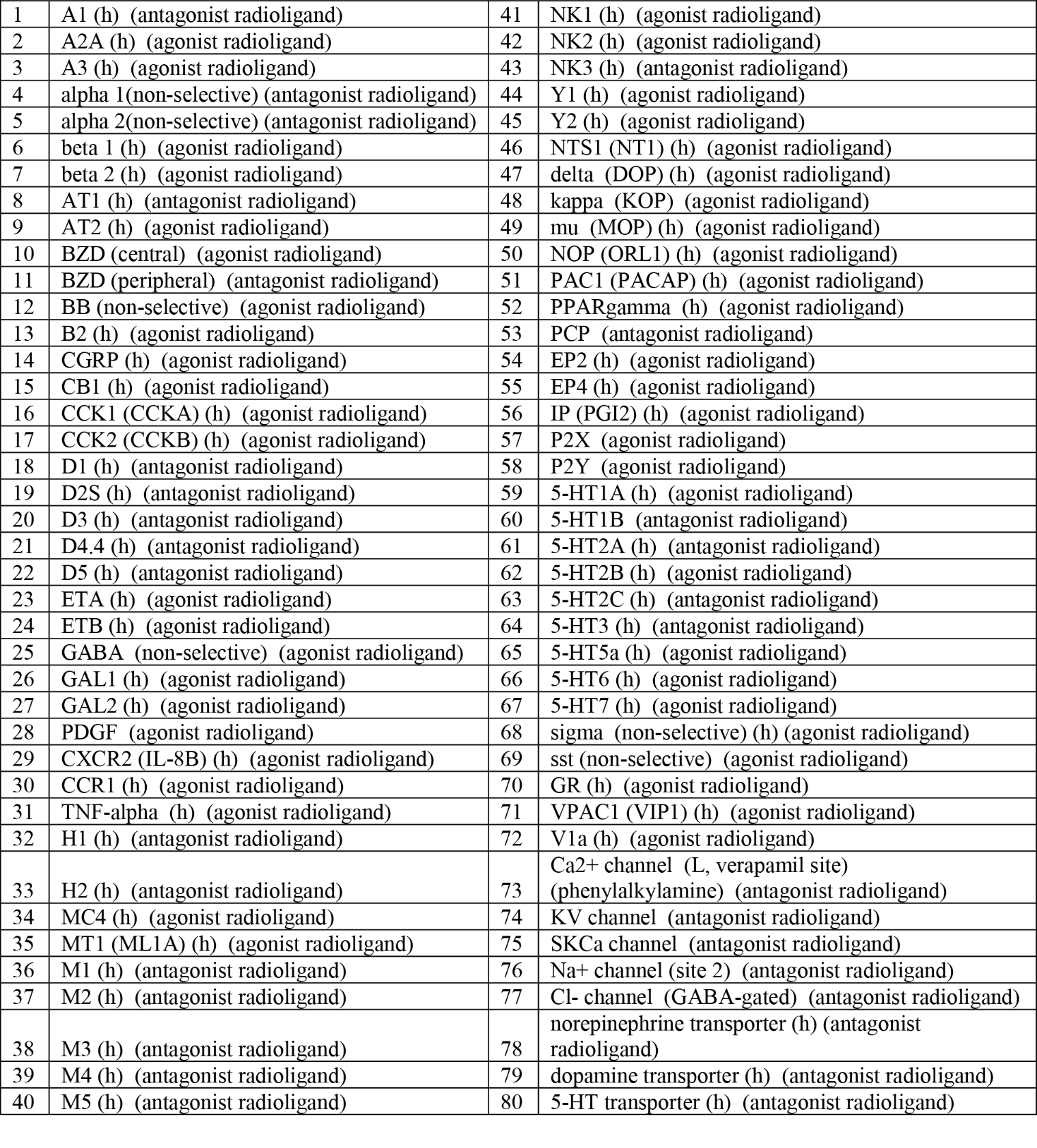


**Supplemental Table 2B: CEREP CNS Panel**


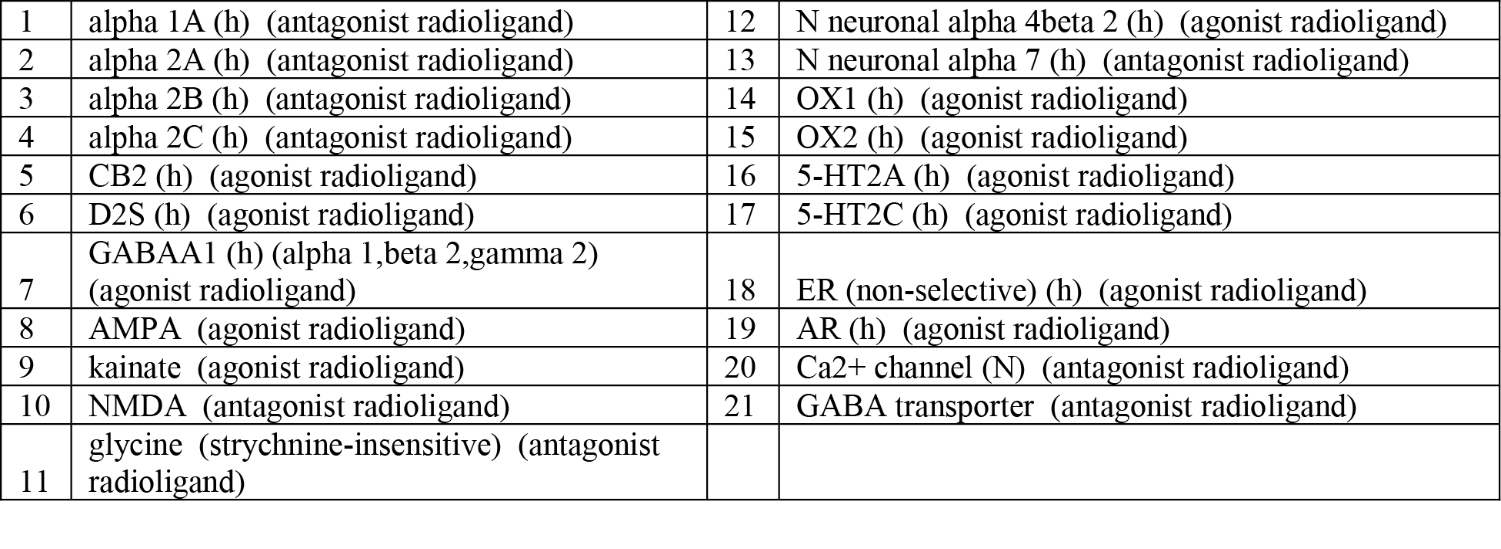


Supplemental Table 2C: Glutamate Receptors Panel


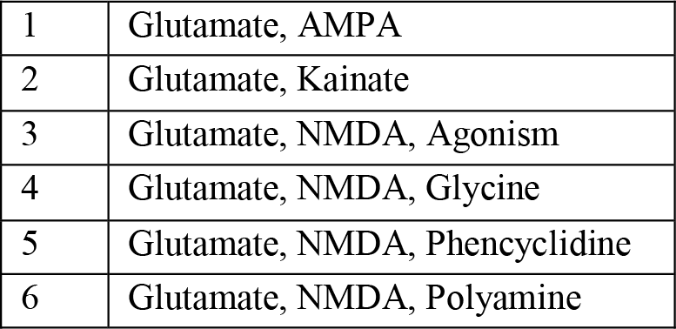


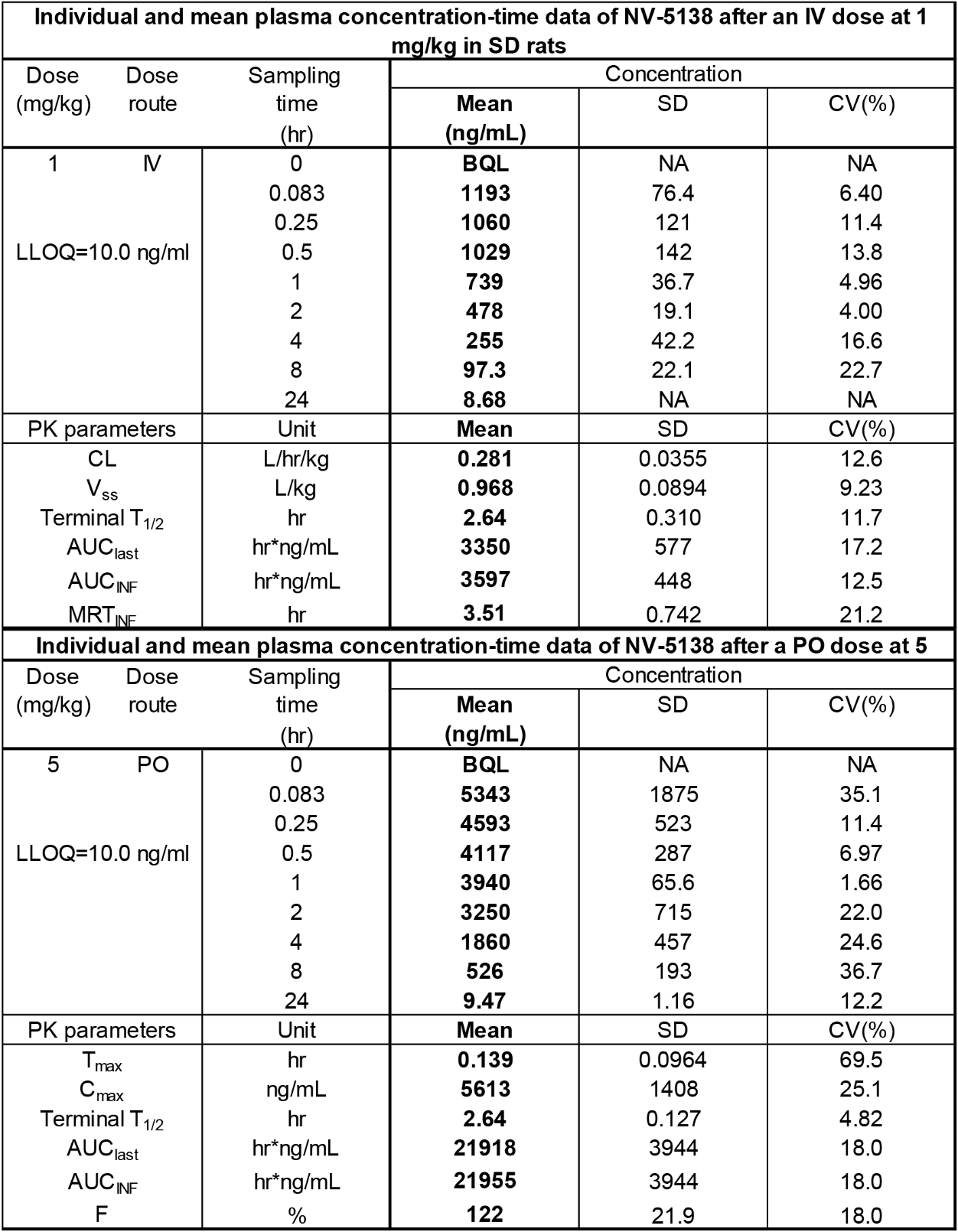


**Supplementary Table 3: Pharmacokinetic analysis of rats dosed with NV-5138 (1 mg/kg, IV and 5 mg/kg PO)**


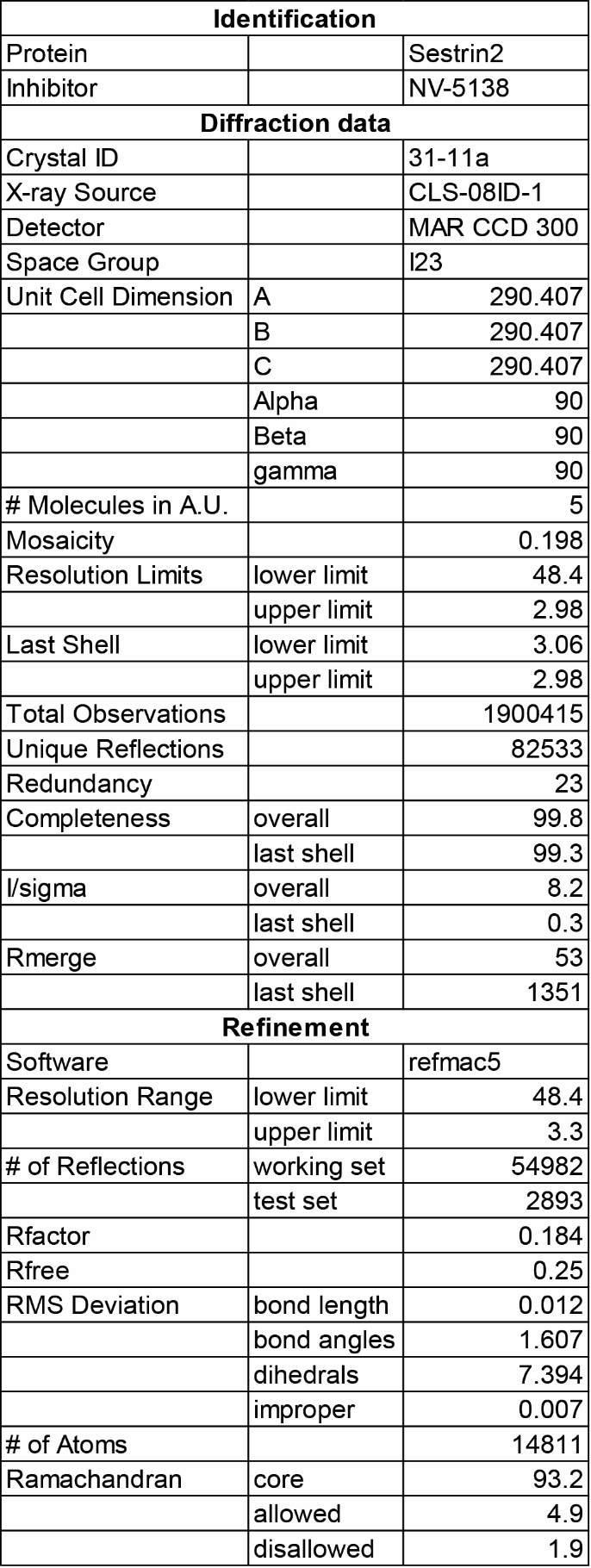


**Supplementary Table 4: Diffraction Data and Refinement Statistics for crystal structure of Sestrin2 bound to NV-5138**

**References**

1 Andersen, K. R., Leksa, N. C. & Schwartz, T. U. Optimized E. coli expression strain LOBSTR eliminates common contaminants from His-tag purification. *Proteins* **81**, 1857-1861, doi:10.1002/prot.24364 (2013).

2 Zhang, G. *et al.* In-depth quantitative proteomic analysis of de novo protein synthesis induced by brain-derived neurotrophic factor. *J Proteome Res* **13**, 5707-5714, doi:10.1021/pr5006982 (2014).
